# CHMP2B promotes CHMP7 mediated nuclear pore complex injury in sporadic ALS

**DOI:** 10.1186/s40478-024-01916-7

**Published:** 2024-12-21

**Authors:** Olivia Keeley, Emma Mendoza, Druv Menon, Alyssa N. Coyne

**Affiliations:** 1 Brain Science Institute, Johns Hopkins University School of Medicine, Baltimore, MD 21205 USA; 2 Department of Neurology, Johns Hopkins University School of Medicine, Baltimore, MD 21205 USA

## Abstract

Alterations to the composition and function of neuronal nuclear pore complexes (NPCs) have been documented in multiple neurodegenerative diseases including Amyotrophic Lateral Sclerosis (ALS). Moreover, recent work has suggested that injury to the NPC can at least in part contribute to TDP-43 loss of function and mislocalization, a pathological hallmark of ALS and related neurodegenerative diseases. Collectively, these studies highlight a role for disruptions in NPC homeostasis and surveillance as a significant pathophysiologic event in neurodegeneration. The ESCRT-III nuclear surveillance pathway plays a critical role in the surveillance and maintenance of NPCs and the surrounding nuclear environment. Importantly, pathologic alterations to this pathway and its protein constituents have been implicated in neurodegenerative diseases such as ALS. However, the mechanism by which this pathway contributes to disease associated alterations in the NPC remains unknown. Here we use an induced pluripotent stem cell (iPSC) derived neuron (iPSN) model of sALS to demonstrate that CHMP7/ESCRT-III nuclear maintenance/surveillance is overactivated in sALS neurons. This overactivation is dependent upon the ESCRT-III protein CHMP2B and sustained CHMP2B dependent “activation” is sufficient to contribute to pathologic CHMP7 nuclear accumulation and POM121 reduction. Importantly, partial knockdown of CHMP2B was sufficient to alleviate NPC injury and downstream TDP-43 dysfunction in sALS neurons thereby highlighting CHMP2B as a potential therapeutic target in disease.

**Supplementary Information:**

The online version contains supplementary material available at 10.1186/s40478-024-01916-7.

## Introduction

Amyotrophic Lateral Sclerosis (ALS) is a neurodegenerative disease that impacts the survival and function of neuronal and glial cells that make up the upper and lower motor circuitry within the brain and spinal cord. About 10% of ALS cases are inherited and thus termed familial ALS (fALS). The remaining 90% of cases are sporadic (sALS) with no known family history. Although mutations in over 20 genes that function in diverse cellular processes including RNA metabolism, protein homeostasis, endolysosomal trafficking and autophagy, and cytoskeletal organization have been linked to fALS and sALS, the majority of sALS cases occur with no known genetic cause [32, 35, 39, 47, 52, 57, 69, 71, 77, 80, 94, 96]. Despite the heterogenous etiology of ALS, 97% of cases display characteristic nuclear depletion and cytoplasmic mislocalization and aggregation of the RNA binding protein TDP-43 in a subset of CNS cells at end-stage disease [9, 15, 25, 31, 61–63]. While the genetics and pathologies of ALS are increasingly well documented, the molecular mechanisms that give rise to altered cellular physiology and pathology, in particular in sALS, remain understudied.

Multiple studies have now documented a role for impaired nuclear-cytoplasmic compartmentalization as a significant contributor to ALS pathophysiology [5, 16, 22, 23, 29, 34, 45, 111]. Recently, patient induced pluripotent stem cell (iPSC) derived neuron (iPSN) models of sALS and C9orf72 ALS/FTD have been utilized to detail an injury to the nuclear pore complex (NPC) that begins with the reduction of the transmembrane nucleoporin (Nup) POM121 as an early and significant contributor to disease [22, 23]. Given that the NPC and its multiple copies of ~ 30 individual Nup constituents collectively and critically control multiple cellular processes including nucleocytoplasmic transport (NCT), gene expression, and genome organization [7, 11, 13, 51, 67, 74, 75, 92], alterations to NPC homeostasis are likely to have detrimental and widespread impacts on neuronal function and survival. In fact, it has been demonstrated that the collective reduction of 8 Nups is sufficient to impact active nuclear import and the localization of mediators of nucleocytoplasmic transport (NCT), the localization and function of TDP-43, and neuronal survival in sALS and C9orf72 ALS/FTD iPSNs [22, 23]. Thus, these studies indicate that altered NPC homeostasis is a critical mediator of pathophysiologic events in sALS.

Recent work has established a fundamental role for the ESCRT-III pathway in the resealing of the nuclear envelope and the surveillance and maintenance of proper NPC assembly, insertion, and function during cell division [30, 36, 64, 95, 105–107]. The ESCRT-III pathway is comprised of multiple protein components including CHMP1-7 and the AAA-ATPase VPS4 and broadly functions in membrane remodeling events that occur during cell division, neuronal pruning, endosomal and exosomal trafficking, and multiple vesicular body formation [58, 103]. Additionally, it has been shown that the ESCRT-III pathway may contribute to the piecemeal turnover and replacement of individual Nups in NPCs in non-neuronal cellular model systems [26, 99, 103, 104]. We have recently demonstrated that the pathologic nuclear accumulation of CHMP7 is sufficient to initiate NPC injury and in turn contribute to TDP-43 dysfunction in sALS iPSNs [23] highlighting alterations to the nuclear surveillance role of the ESCRT-III pathway as a contributor to neurodegenerative pathophysiology. However, ESCRT-III proteins do not typically function as single protein units and pathway function often proceeds following coordinated recruitment and ESCRT protein “activation” (e.g. removal of autoinhibition) and polymerization. This activation step is achieved via protein – protein interactions and can occur between ESCRT-III proteins themselves [58, 103]. As such, the molecular mechanisms that give rise to CHMP7 mediated NPC injury and the involvement of ESCRT-III protein partners in this process remains unknown.

In this study, we demonstrate that the ESCRT-III protein CHMP2B play a central role in eliciting CHMP7 mediated NPC injury in sALS iPSNs. Specifically, CHMP2B facilitates the “activation” of CHMP7 within the nucleus thereby triggering the reduction of POM121 at the early stages of NPC injury cascades. Interestingly, sustained CHMP2B mediated activation appears to underlie the pathologic nuclear accumulation/retention of CHMP7 observed in sALS. Critically, moderate reduction of CHMP2B protein via antisense oligonucleotide (ASO) or siRNA approaches abrogates nuclear accumulation of CHMP7, restores the nuclear localization and expression of POM121, and alleviates TDP-43 dysfunction in sALS iPSNs. Thus, these data define a cell biological mechanism underlying CHMP7 nuclear accumulation and subsequent initiation of NPC injury in the pathogenesis of sALS. Moreover, our study highlights the potential of targeting CHMP2B itself of CHMP7 – CHMP2B protein interactions as a therapeutic strategy for sALS.

## Results

### CHMP7/ESCRT-III mediated Nup turnover is over-active in sALS iPSNs

Prior studies using bulk mass spec based analyses have demonstrated that many, but not all, Nup proteins long-lived within the CNS [83, 98]. Thus, it is thought that entire new NPCs are not inserted into the nuclear envelope following initial assembly in non-dividing neurons. However, the NPC is a highly organized octet structure comprised of multiple copies of individual Nup protein molecules [8, 51, 65, 70, 76] which display differing residence times within NPCs [37]. Thus, within a given NPC, it is likely that individual molecules of each Nup protein are turned over at varying rates in order to maintain NPC homeostasis. Indeed, recent evidence has demonstrated that Nup96, Nup93, Nup133, and POM121 Nup molecules are turned over within myoblast and myotube NPCs are varying rates with many NPCs containing new and old Nup molecules within 1–2 weeks [99].

Among many cellular functions, the ESCRT-III pathway plays a fundamental role in maintaining nuclear envelope and NPC homeostasis. To ensure nuclear envelope sealing and proper insertion and assembly of NPCs during cell division, the ESCRT-III protein CHMP7 passively translocates from the cytoplasm to the nucleus where it engages in specific protein – protein interactions to initiate its “activation” and the subsequent recruitment and polymerization of additional ESCRT-III proteins. Ultimately, this results in removal of improperly assembled NPCs and/or sealing of the nuclear envelope [30, 36, 64, 68, 95, 105, 107]. In addition, recent studies have proposed a role for ESCRT-III proteins in the piecemeal turnover of individual Nups within existing NPCs in non-dividing non-neuronal cells [99]. Thus, these data support a fundamental role for the ESCRT-III pathway in the maintenance of NPCs throughout the lifetime of cells. However, whether the CHMP7/ESCRT-III nuclear surveillance pathway plays a role in maintenance of NPCs throughout the lifetime of human neurons remains unknown.

**Fig. 1:**
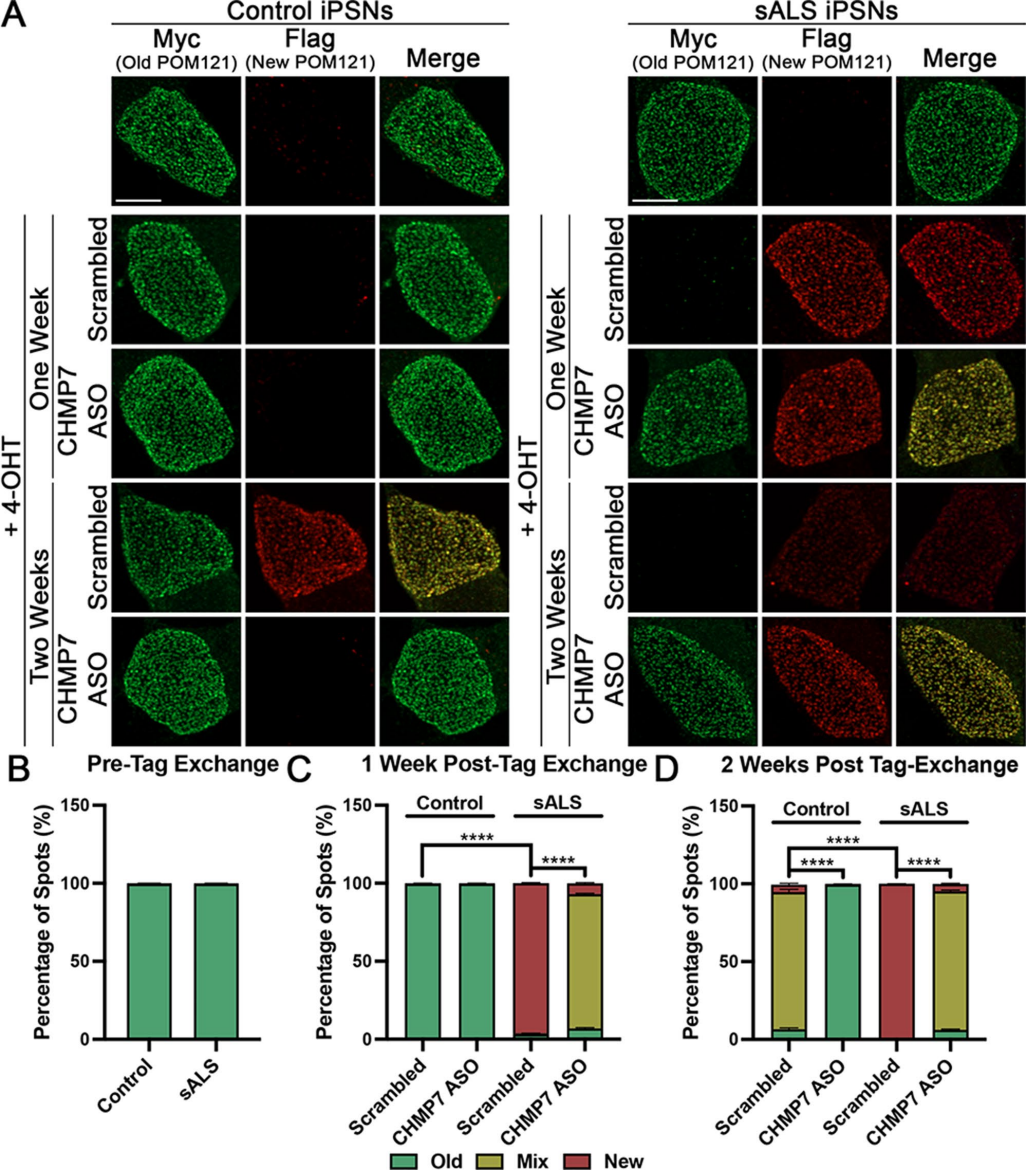
CHMP7 mediated Nup turnover is overactive in sALS iPSNs. (**A**) Maximum intensity projections from Airyscan imaging of Myc (Old) and Flag (New) tagged POM121 in control and sALS iPSN nuclei. POM121 RITE plasmids were expressed starting at day 15 of differentiation. Scrambled control and CHMP7 targeting ASO treatment was initiated at Day 19 of differentiation. 4-OHT treatment was initiated at day 22 of differentiation. Treatment and time point as indicated on left. (**B-D**) Quantification of old, mixed, and new spots pre tag exchange (**B**) and one (**C**) and two (**D**) weeks post tag exchange. *n* = 6 control and 6 sALS iPSC lines, 50 iPSNs per line/treatment. Fisher’s exact was used to calculate statistical significance. **** *p* < 0.0001. Scale bar = 5 μm

We have previously shown that NPC injury cascades begin with the reduction of POM121 from ALS neuronal NPCs [22]. Thus, preservation of POM121 within NPCs of human neurons is critical for maintenance of NPC composition and function to prevent or reverse downstream pathophysiologic events [21, 46, 82]. Given our prior demonstration that the nuclear accumulation of CHMP7 is sufficient to drive aberrant and pathologic reduction of specific Nups from the neuronal NPC [23], we hypothesized that the CHMP7/ESCRT-III nuclear surveillance pathway may be overactive in sALS thereby facilitating aberrant Nup molecule/protein turnover to give rise to this documented NPC injury. To begin to test this hypothesis, we utilized Recombination Induced Tag Exchange (RITE) technology [102] to monitor new and old POM121 expression within control and sALS. Upon initial expression of a previously detailed POM121 RITE plasmid [99], exogenous POM121 can be visualized with antibodies against a Myc tag. Following addition of 4-hydroxytamoxifen (4-OHT) to induce tag replacement via the loxP-Cre recombinase system, newly synthesized POM121 molecules can be visualized with antibodies against a Flag tag. Thus, old POM121 proteins will be labeled with Myc and new POM121 proteins will be labeled with Flag. When combined with Airyscan imaging which provides increased imaging resolution necessary to visualize individual NPC spots [90, 109], this methodology affords the opportunity to examine POM121 protein turnover within iPSN NPCs, albeit not at the resolution of individual Nup molecules within NPCs. NPCs in control iPSNs exclusively contained “old” POM121 protein until 2 weeks post tag-exchange induction, at which point NPCs were predominantly composed of a mixture of “new” and “old” POM121 proteins (Fig. 1). As imaging technologies that enable an evaluation of the number of POM121 molecules exchanged in individual NPCs are not yet available for iPSNs, we are unable to determine the precise number of individual POM121 molecules exchanged per iPSN NPC at this time. Thus, although most NPCs contained both “new” and “old” POM121 molecules at this time point, it is possible that each NPC contains a variable number of “new” POM121 molecules that cannot be distinguished by signal intensities at this resolution. Nonetheless, these data suggest that a substantial number of POM121 molecules per NPC are likely exchanged at a rate of about every 2 weeks in cultured iPSNs. In contrast, by 1 week post tag-exchange induction, sALS NPCs were comprised almost exclusively of “new” POM121 protein (Fig. 1). These data suggest that POM121 protein replenishment occurs much more frequently in sALS iPSN NPCs. Consistent with our prior publication detailing a pathological reduction of POM121 in sALS iPSNs [23], by 2 weeks post-tag exchange induction, even “new” POM121 protein appeared to be partially depleted from sALS iPSNs (Fig. 1).

To examine whether the CHMP7/ESCRT-III nuclear surveillance pathway functioned in the fundamental turnover/replenishment of POM121 protein in iPSNs, we employed antisense oligonucleotides (ASOs) [23] to reduce CHMP7 and examined “new” vs “old’ POM121 protein in iPSN NPCs by Airyscan microscopy. ASO mediated knockdown of CHMP7 was sufficient to prevent the incorporation of “new” POM121, thereby retaining “old” POM121 within NPCs and slowing POM121 turnover in control iPSNs (Fig. 1). In addition, reduction of CHMP7 resulted in a partial retention of “old” POM121 one week following induction of tag-exchange in sALS iPSNs (Fig. 1) indicative of slowed POM121 turnover. This mixed expression of “old” and “new” POM121 within sALS iPSN NPCs was maintained 2 weeks post induction of tag-exchange (Fig. 1). Thus, pathologic CHMP7/ESCRT-III mediated reduction of POM121 was prevented consistent with our prior report that knockdown of CHMP7 could restore the localization and expression of POM121 within ALS NPCs [23]. Together, these data suggest that the fundamental function of the CHMP7/ESCRT-III nuclear surveillance pathway in facilitating piecemeal Nup turnover within neuronal NPCs is “overactive” in sALS thereby giving rise to pathologic Nup reduction.

### CHMP2B promotes the nuclear retention of CHMP7 in sALS iPSNs

**Fig. 2:**
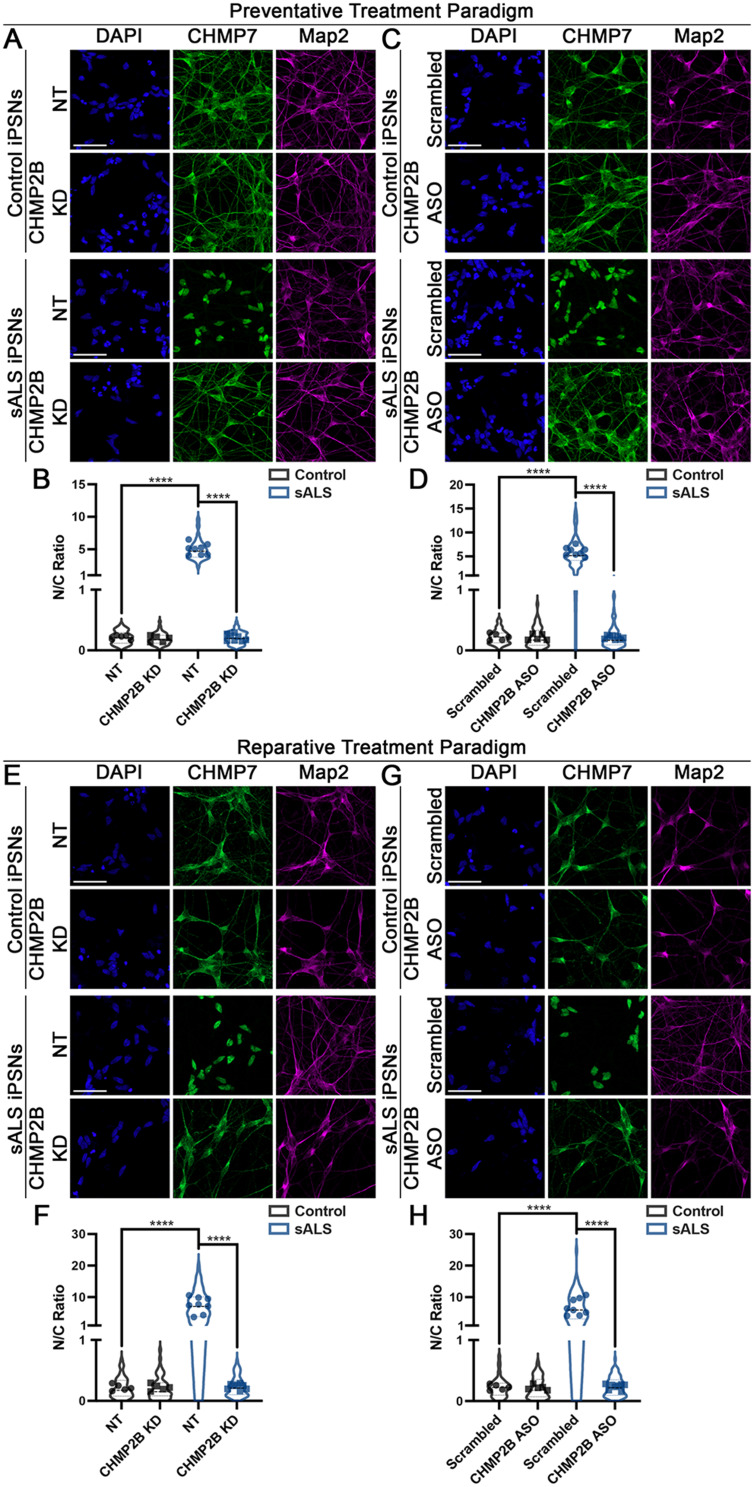
Reduction of CHMP2B is sufficient to alleviate increased nuclear localization of CHMP7 in sALS iPSNs. (**A**) Maximum intensity projections from immunostaining and confocal imaging of CHMP7 in control and sALS iPSNs on day 46 of differentiation (3 weeks following nucleofection of CHMP2B or non-targeting (NT) siRNAs). siRNA and genotype as indicated on left, antibodies for immunostaining as indicated on top. Knockdown was initiated at day 15 of differentiation (the time point where nuclear localization of CHMP7 begins to increase in sALS iPSNs [23]). (**B**) Quantification of nuclear to cytoplasmic ratios of CHMP7. *n* = 5 control and 8 sALS iPSC lines, 100 Map2 + cells per line/knockdown. Two-way ANOVA with Tukey’s multiple comparison test was used to calculate statistical significance. **** *p* < 0.0001. (**C**) Maximum intensity projections from immunostaining and confocal imaging of CHMP7 in control and sALS iPSNs on day 46 of differentiation following 3 weeks of treatment with scrambled control of CHMP2B targeting ASOs. ASO and genotype as indicated on left, antibodies for immunostaining as indicated on top. ASO treatment was initiated at day 15 of differentiation (the time point where nuclear localization of CHMP7 begins to increase in sALS iPSNs [23]). (**D**) Quantification of nuclear to cytoplasmic ratios of CHMP7. *n* = 5 control and 8 sALS iPSC lines, 100 Map2 + cells per line/treatment. Two-way ANOVA with Tukey’s multiple comparison test was used to calculate statistical significance. **** *p* < 0.0001. (**E**) Maximum intensity projections from immunostaining and confocal imaging of CHMP7 in control and sALS iPSNs on day 81 of differentiation (3 weeks following nucleofection of CHMP2B or non-targeting (NT) siRNAs). siRNA and genotype as indicated on left, antibodies for immunostaining as indicated on top. Knockdown was initiated at day 60 of differentiation following the emergence of NPC injury and TDP-43 loss of function and mislocalization [23, 82]. (**F**) Quantification of nuclear to cytoplasmic ratios of CHMP7. *n* = 5 control and 8 sALS iPSC lines, 100 Map2 + cells per line/knockdown. Two-way ANOVA with Tukey’s multiple comparison test was used to calculate statistical significance. **** *p* < 0.0001. (**G**) Maximum intensity projections from immunostaining and confocal imaging of CHMP7 in control and sALS iPSNs on day 81 of differentiation following 3 weeks of treatment with scrambled control of CHMP2B targeting ASOs. ASO and genotype as indicated on left, antibodies for immunostaining as indicated on top. ASO treatment was initiated at day 60 of differentiation following the emergence of NPC injury and TDP-43 loss of function and mislocalization [23, 82]. (**H**) Quantification of nuclear to cytoplasmic ratios of CHMP7. *n* = 5 control and 8 sALS iPSC lines, 100 Map2 + cells per line/treatment. Two-way ANOVA with Tukey’s multiple comparison test was used to calculate statistical significance. **** *p* < 0.0001. Scale bar = 50 μm

Typically, individual ESCRT-III proteins do not function in isolation. Instead, they function as a unit with other pathway constituents [58, 103]. We have previously demonstrated that the nuclear expression and localization of two of the more well studied ESCRT-III proteins CHMP2B and CHMP4B are not pathologically altered in sALS [20]. However, given the basal nuclear distribution of ESCRT-III proteins in iPSNs, we hypothesized that perhaps they could be functionally relevant for disease associated alterations in CHMP7/ESCRT-III mediated nuclear surveillance pathway pathology and function. For this present study, we elected to focus on the role of CHMP2B given that mutations in CHMP2B have been implicated in Frontotemporal Dementia (FTD) and a lower motor neuron predominant form of ALS [18, 19, 66, 79, 91, 100]. Given our data that CHMP7 dependent Nup turnover is “over-active” in sALS iPSNs (Fig. 1) and CHMP7 is abnormal accumulated within sALS nuclei [23], we first wanted to test whether CHMP2B was functionally implicated in eliciting CHMP7 pathology in sALS. To do this, we used 2 independent strategies, siRNA (Supplemental Fig. S1) and antisense oligonucleotides (Supplemental Fig. S2) to knockdown human CHMP2B in iPSNs. In addition, we utilized two treatment paradigms (Supplemental Fig. S3). The first is a “preventative treatment” paradigm where knockdown was initiated prior to the emergence of nuclear accumulation of CHMP7 (Supplemental Fig. S3A) to facilitate an analysis of the function of CHMP2B in promoting CHMP7 pathology in sALS. The second is a “reparative treatment” where knockdown was initiated following the emergence of multiple NPC injury associated events (Supplemental Fig. S3B) to mimic a clinically relevant scenario where patients received treatment following symptom onset. Immunostaining and confocal imaging for CHMP7 demonstrated that 3 weeks of sustained ~ 50% reduction of endogenous CHMP2B (Supplemental Figs. S1–S2) was sufficient to both prevent and reverse the pathologic nuclear accumulation of CHMP7 in sALS iPSNs (Fig. 2, see Supplemental Fig. S4 for high magnification images). We did not observe any difference in the magnitude of prevention with either siRNA or ASO (Fig. 2). Thus, these data suggest that CHMP2B plays a critical role in promoting the aberrant nuclear accumulation/retention of CHMP7 observed in sALS.

### Knockdown of CHMP2B alleviates NPC injury cascades in sALS iPSNs

**Fig. 3:**
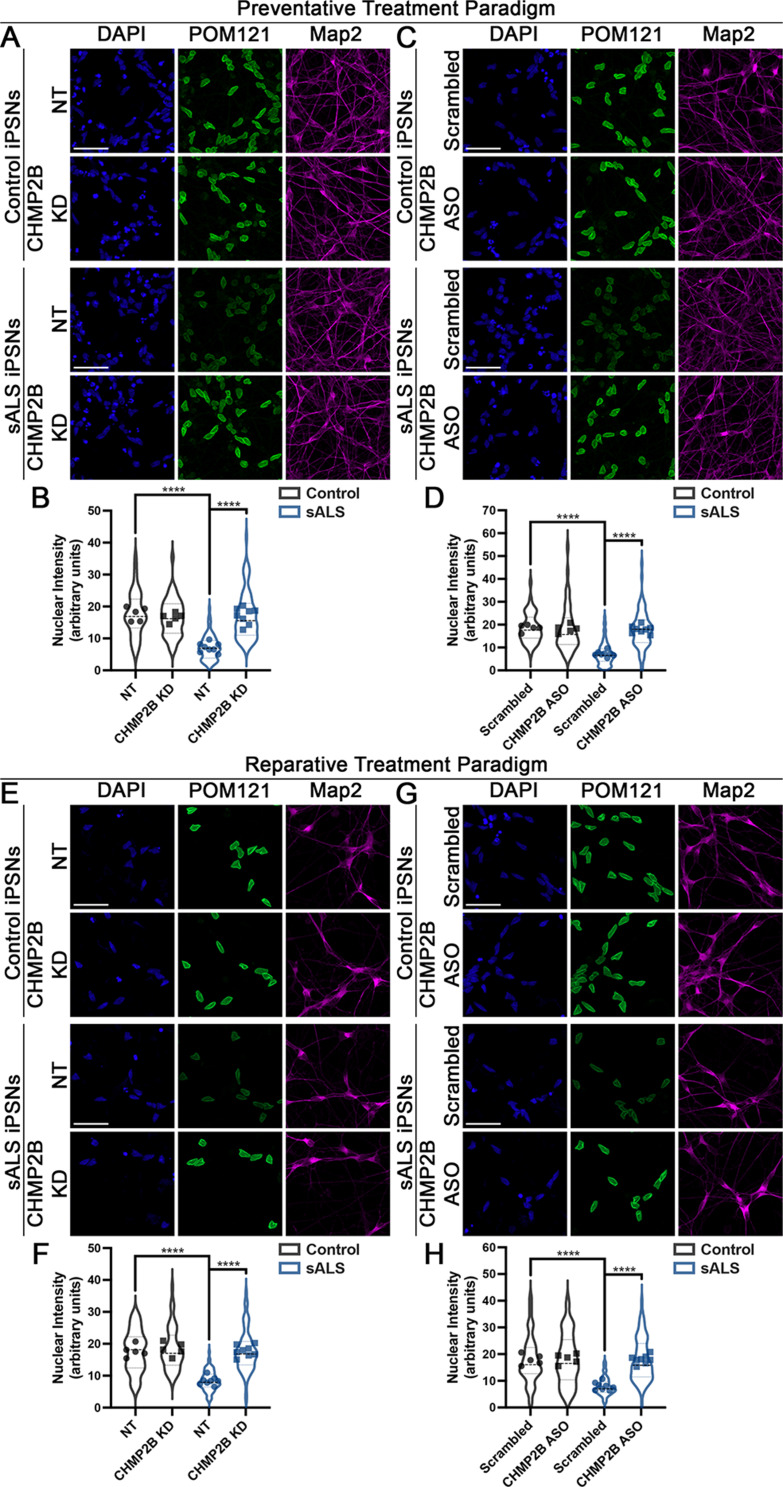
Reduction of CHMP2B is sufficient to mitigate reduction of nuclear POM121 in sALS iPSNs. (**A**) Maximum intensity projections from immunostaining and confocal imaging of POM121 in control and sALS iPSNs on day 46 of differentiation (3 weeks following nucleofection of CHMP2B or non-targeting (NT) siRNAs). siRNA and genotype as indicated on left, antibodies for immunostaining as indicated on top. Knockdown was initiated at day 15 of differentiation (the time point where nuclear localization of CHMP7 begins to increase in sALS iPSNs [23]). (**B**) Quantification of POM121 nuclear intensity. *n* = 5 control and 8 sALS iPSC lines, 100 Map2 + cells per line/knockdown. Two-way ANOVA with Tukey’s multiple comparison test was used to calculate statistical significance. **** *p* < 0.0001. (**C**) Maximum intensity projections from immunostaining and confocal imaging of POM121 in control and sALS iPSNs on day 46 of differentiation following 3 weeks of treatment with scrambled control of CHMP2B targeting ASOs. ASO and genotype as indicated on left, antibodies for immunostaining as indicated on top. ASO treatment was initiated at day 15 of differentiation (the time point where nuclear localization of CHMP7 begins to increase in sALS iPSNs [23]). (**D**) Quantification of POM121 nuclear intensity. *n* = 5 control and 8 sALS iPSC lines, 100 Map2 + cells per line/treatment. Two-way ANOVA with Tukey’s multiple comparison test was used to calculate statistical significance. **** *p* < 0.0001. (**E**) Maximum intensity projections from immunostaining and confocal imaging of POM121 in control and sALS iPSNs on day 81 of differentiation (3 weeks following nucleofection of CHMP2B or non-targeting (NT) siRNAs). siRNA and genotype as indicated on left, antibodies for immunostaining as indicated on top. Knockdown was initiated at day 60 of differentiation following the emergence of NPC injury and TDP-43 loss of function and mislocalization [23, 82]. (**F**) Quantification of POM121 nuclear intensity. *n* = 5 control and 8 sALS iPSC lines, 100 Map2 + cells per line/knockdown. Two-way ANOVA with Tukey’s multiple comparison test was used to calculate statistical significance. **** *p* < 0.0001. (**G**) Maximum intensity projections from immunostaining and confocal imaging of POM121 in control and sALS iPSNs on day 81 of differentiation following 3 weeks of treatment with scrambled control of CHMP2B targeting ASOs. ASO and genotype as indicated on left, antibodies for immunostaining as indicated on top. ASO treatment was initiated at day 61 of differentiation following the emergence of NPC injury and TDP-43 loss of function and mislocalization [23, 82]. (**H**) Quantification of POM121 nuclear intensity. *n* = 5 control and 8 sALS iPSC lines, 100 Map2 + cells per line/treatment. Two-way ANOVA with Tukey’s multiple comparison test was used to calculate statistical significance. **** *p* < 0.0001. Scale bar = 50 μm

**Fig. 4:**
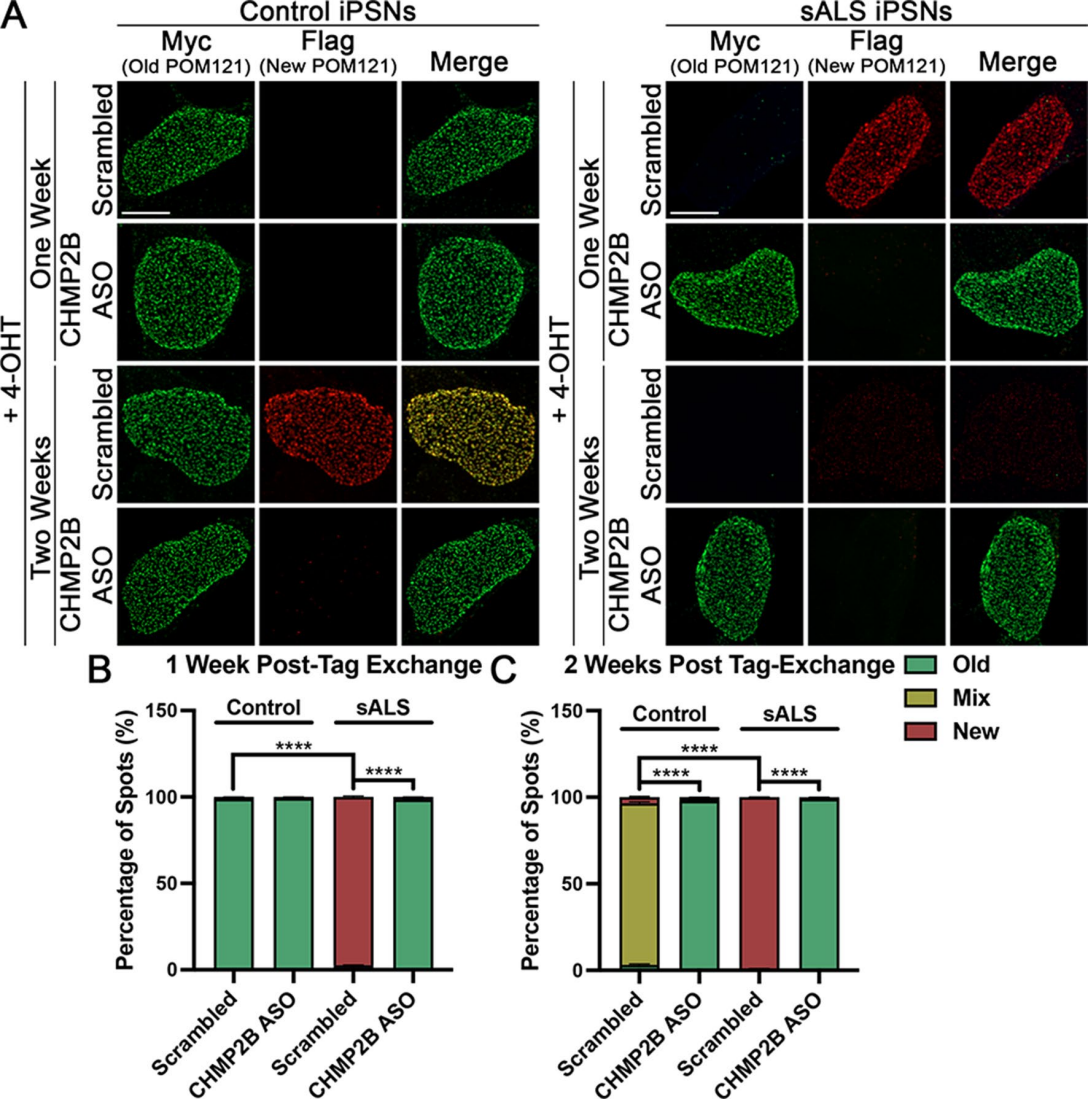
Reduction of CHMP2B diminishes turnover of POM121 in iPSNs. (**A**) Maximum intensity projections from Airyscan imaging of Myc (Old) and Flag (New) tagged POM121 in control and sALS iPSN nuclei. POM121 RITE plasmids were expressed starting at day 15 of differentiation. Scrambled control or CHMP2B targeting ASO treatment was initiated at day 19 of differentiation. 4-OHT treatment was initiated at day 22 of differentiation. Treatments and time point as indicated on left. (**B-C**) Quantification of old, mixed, and new spots one (**B**) and two (**C**) weeks post tag exchange. *n* = 6 control and 6 sALS iPSC lines, 50 iPSNs per line/treatment. Fisher’s exact was used to calculate statistical significance. **** *p* < 0.0001. Scale bar = 5 μm

We have recently shown that nuclear accumulation of CHMP7 can trigger the reduction of specific Nups from the nucleus in ALS iPSNs [23]. Given that reduction of CHMP2B alleviates nuclear accumulation of CHMP7 in sALS iPSNs (Fig. 2, Supplemental Fig. S4), we next asked whether knockdown of CHMP2B was sufficient to alleviate NPC injury in sALS. Similar to our recent study [5], we elected to use POM121 as an indicator of NPC injury as we have previously demonstrated that reduction of POM121 is one of the earliest pathological events within the NPC itself in ALS iPSNs [22, 82]. Immunostaining and confocal imaging for POM121 demonstrated that reduction of CHMP2B by either ASO or siRNA significantly increased nuclear POM121 immunoreactivity in sALS iPSNs to levels observed in control iPSNs (Fig. 3, see Supplemental Fig. S5 for high magnification images). Nuclei isolation and western blotting supported this imaging-based observation (Supplemental Fig. S6). Importantly, this restorative effect was observed using both preventative and reparative siRNA and ASO treatment paradigms (Fig. 3, Supplemental Figs. S5–S6). Next, we utilized our POM121 RITE plasmid to examine the impact of reduced CHMP2B expression on the turnover of POM121 in sALS iPSN nuclei and NPCs. Airyscan imaging revealed a significant decrease in the incorporation of “new” POM121 within both control and sALS iPSN nuclei post induction of tag exchange (Fig. 4). Interestingly, this sustainment of “old” POM121 expression appeared to be stronger than the effect of CHMP7 knockdown (Figs. 1 and 4) suggesting that CHMP2B may function upstream of CHMP7 in ESCRT-III mediated Nup turnover in human neurons. Taken together, these data functionally implicate CHMP2B in the initiation of overactive Nup turnover and ESCRT-III/CHMP7 mediated NPC injury in sALS.

In neurodegenerative diseases such as ALS, studies in autopsy tissues have documented an altered nucleocytoplasmic distribution of the RNA binding protein TDP-43. While typically primarily localized to the nucleus, in disease, TDP-43 becomes mislocalized to the cytoplasm where in a small subset of cells it can subsequently assemble into cytoplasmic aggregated. Although detectable in the vast majority of ALS autopsies, the extent to which the subcellular distribution of TDP-43 is altered in individual neurons is extremely variable [4, 9, 15, 25, 31, 44, 50, 61–63, 101]. Nonetheless, the varying degrees of nuclear depletion are thought to induce a nuclear loss of TDP-43 function that results in detectable and quantifiable alterations in gene expression as well as inclusion of cryptic exons within mRNAs which is one hallmark of alterations in splicing [10, 40, 43, 48, 53, 55, 59, 72, 73, 82, 85]. We have recently demonstrated that although these molecular signatures of TDP-43 dysfunction are variable, they are detectable amongst individual sALS patient iPSNs [82]. Moreover, we have demonstrated that CHMP7 mediated NPC injury is a significant contributor to TDP-43 dysfunction in ALS iPSNs [23, 82]. As a result, we selected a subset of 14 gene expression and mRNA splicing alterations previously established to occur following artificial TDP-43 depletion in human neurons [5, 10, 48, 85] to monitor “TDP-43 function” via qRT-PCR following CHMP2B reduction in sALS iPSNs. In doing so, we found that both siRNA and ASO mediated reduction of CHMP2B significantly prevented the TDP-43 loss of function events (Supplemental Figs. S7–S8 A-N) and restored TDP-43 function in sALS iPSNs (Supplemental Figs. S9–S10 A-N). Importantly, knockdown of CHMP2B had no impact on the expression of 2 mRNAs, *ACTIN* and *POM121* that are not thought to be regulated by TDP-43 (Supplemental Figs. S7–S10O-P). Consistent with a prevention of TDP-43 loss of function or restoration of TDP-43 function, both siRNA and ASO mediated CHMP2B reduction also prevented and reversed the subtle TDP-43 mislocalization observed in sALS iPSNs (Supplemental Figs. S7–S10Q-S). Thus, in aggregate, these data highlight CHMP2B as a potent mediator of CHMP7 mediated NPC injury cascades in sALS.

### Knockdown of CHMP2B mitigates glutamate induced neuronal death in sALS iPSNs

**Fig. 5:**
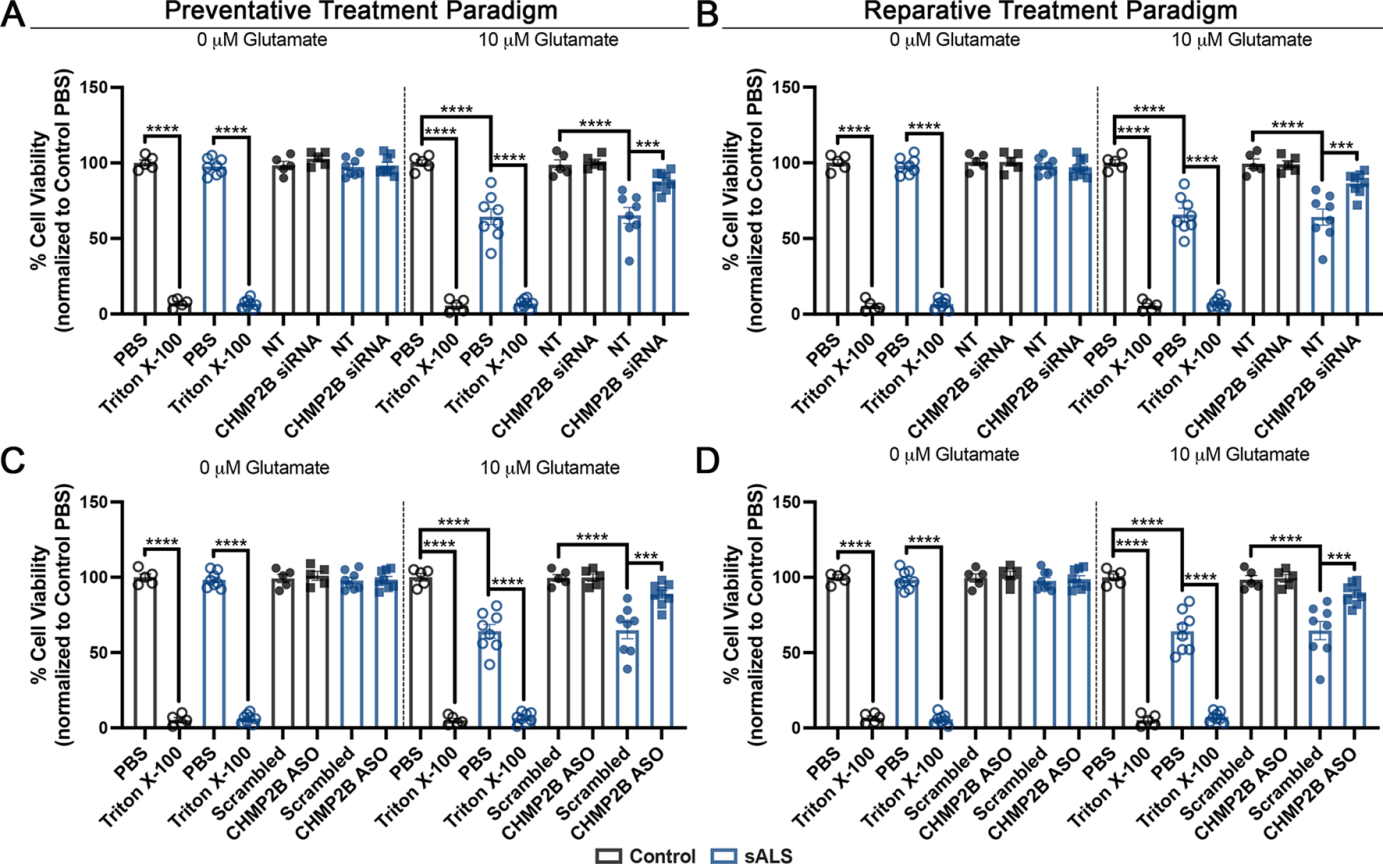
siRNA and ASO mediated reduction of CHMP2B increases viability of sALS iPSNs following exposure to excess glutamate. (**A**) Percent cell viability as measured by Alamar Blue in control and sALS iPSNs following 4 h exposure to 0 or 10 µM glutamate on day 46 of differentiation (3 weeks following nucleofection of CHMP2B or non-targeting (NT) siRNAs). Knockdown was initiated at day 15 of differentiation (the time point where nuclear localization of CHMP7 begins to increase in sALS iPSNs [23]). PBS was used for normalization. Triton X-100 was used as a positive control to induce neuronal death. *n* = 5 control and 8 sALS iPSC lines. Data points represent the average percent viability from 3 technical replicate wells for each line/siRNA. Two-way ANOVA with Tukey’s multiple comparison test was used to calculate statistical significance. *** *p* < 0.001, **** *p* < 0.0001. (**B**) Percent cell viability as measured by Alamar Blue in control and sALS iPSNs following 4 h exposure to 0 or 10 µM glutamate on day 46 of differentiation following 3 weeks of treatment with scrambled control of CHMP2B targeting ASOs. ASO treatment was initiated at day 15 of differentiation (the time point where nuclear localization of CHMP7 begins to increase in sALS iPSNs [23]). PBS was used for normalization. Triton X-100 was used as a positive control to induce neuronal death. *n* = 5 control and 8 sALS iPSC lines. Data points represent the average percent viability from 3 technical replicate wells for each line/ASO. Two-way ANOVA with Tukey’s multiple comparison test was used to calculate statistical significance. *** *p* < 0.001, **** *p* < 0.0001. (**C**) Percent cell viability as measured by Alamar Blue in control and sALS iPSNs following 4 h exposure to 0 or 10 µM glutamate on day 81 of differentiation (3 weeks following nucleofection of CHMP2B or non-targeting (NT) siRNAs). Knockdown was initiated at day 60 of differentiation following the emergence of NPC injury and TDP-43 loss of function and mislocalization [23, 82]. PBS was used for normalization. Triton X-100 was used as a positive control to induce neuronal death. *n* = 5 control and 8 sALS iPSC lines. Data points represent the average percent viability from 3 technical replicate wells for each line/siRNA. Two-way ANOVA with Tukey’s multiple comparison test was used to calculate statistical significance. *** *p* < 0.001, **** *p* < 0.0001. (**D**) Percent cell viability as measured by Alamar Blue in control and sALS iPSNs following 4 h exposure to 0 or 10 µM glutamate on day 81 of differentiation following 3 weeks of treatment with scrambled control of CHMP2B targeting ASOs. ASO treatment was initiated at day 60 of differentiation following the emergence of NPC injury and TDP-43 loss of function and mislocalization [23, 82]. PBS was used for normalization. Triton X-100 was used as a positive control to induce neuronal death. *n* = 5 control and 8 sALS iPSC lines. Data points represent the average percent viability from 3 technical replicate wells for each line/ASO. Two-way ANOVA with Tukey’s multiple comparison test was used to calculate statistical significance. *** *p* < 0.001, **** *p* < 0.0001

Having established that partial reduction of CHMP2B could protect against and reverse multiple pathophysiologic events in NPC injury cascades in sALS iPSNs (Figs. 2 and 3, Supplemental Figs. 4–10), we next asked whether knockdown of CHMP2B was sufficient to increase neuronal survival. Consistent with our prior publications [5, 22, 23, 81], we did not detect basal levels of cell death in our sALS iPSN cultures (Fig. 5, Supplemental Fig. S11). However, as we and others have previously demonstrated [5, 22, 23, 28], sALS iPSNs were sensitive to glutamate induced cell death as assessed by independent measures of cell death: Alamar Blue cell viability (Fig. 5) and propidium idodide uptake (Supplemental Fig. S11). This phenomenon was both prevented and reversed by treatment with CHMP2B targeting siRNAs and ASOs (Fig. 5, Supplemental Fig. S11). Importantly, partial reduction of CHMP2B was not overtly toxic to control iPSNs and had no negative impact on neuronal survival in the absence of glutamate induced cell death (Fig. 5, Supplemental Fig. S11). Together, these data support a protective role for partial reduction of CHMP2B in sALS iPSNs.

### CHMP7 and CHMP2B persistently associate in close proximity in sALS iPSN nuclei

**Fig. 6:**
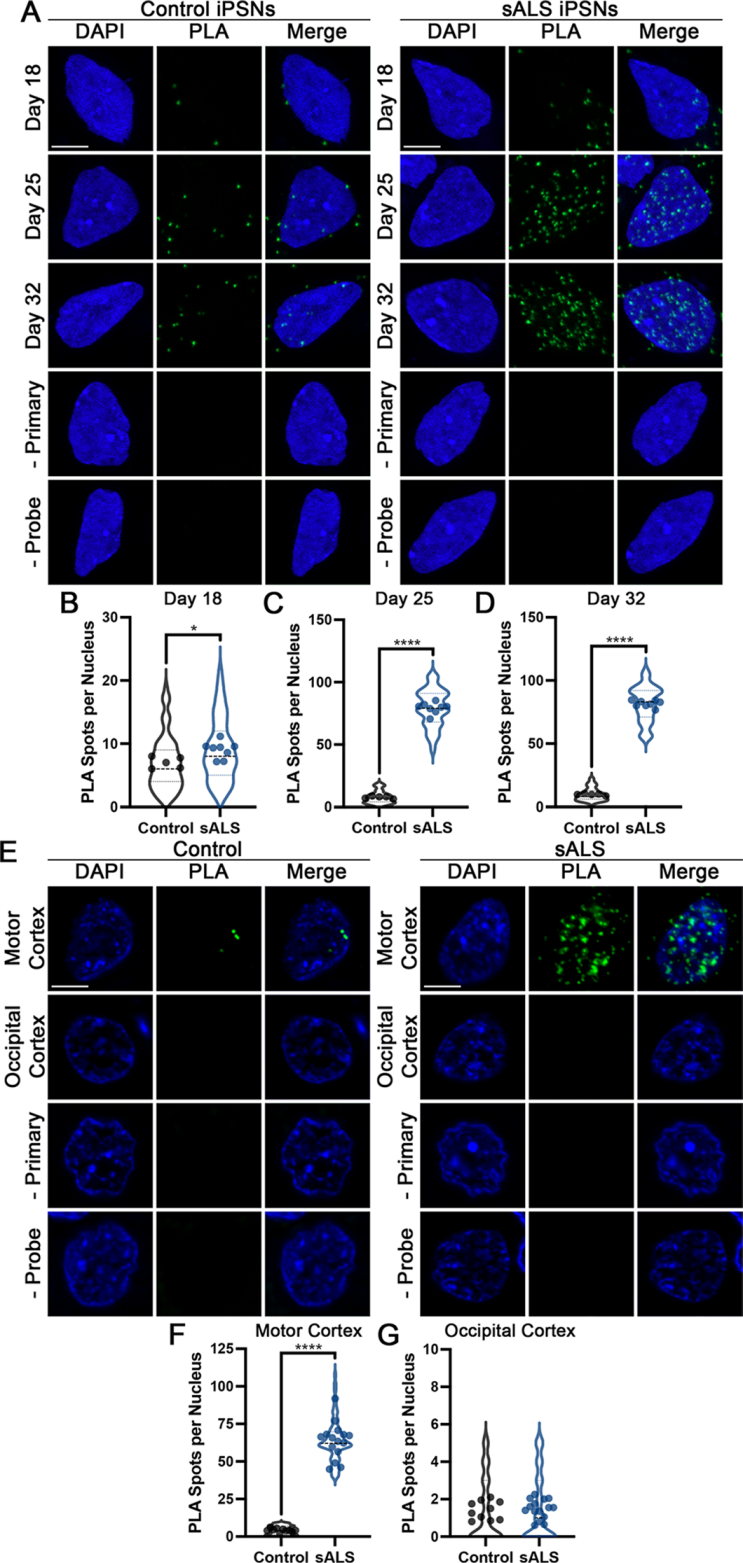
The proximity between CHMP7 and CHMP2B is increased and sustained in sALS nuclei. (**A**) Maximum intensity projections from confocal imaging of CHMP7 – CHMP2B PLA signals in control and sALS iPSNs. Time points as indicated on left, genotype as indicated on top. (**B-D**) Quantification of number of PLA signals per nucleus at day 18 (**B**), day 25 (**C**) and day 32 (**D**). *n* = 5 control and 8 sALS iPSC lines, 100 nuclei per line/timepoint. Student’s t-test was used to calculate statistical significance. * *p* < 0.05, **** *p* < 0.0001. (**E**) Single z sections from apotome based imaging of CHMP7 – CHMP2B PLA signals in control and sALS postmortem layer V motor and occipital cortex tissue. Brain region as indicated on left, genotype as indicated on top. (**F-G**) Quantification of number of PLA signals per nucleus in motor (**F**) and occipital (**G**) cortex tissue. *n* = 10 control and 15 sALS cases, 100 nuclei per patient/brain region. Student’s t-test was used to calculate statistical significance. **** *p* < 0.0001. Scale bar = 5 μm

ESCRT-III proteins often interact in multi-protein complexes to facilitate polymerization and function [58, 84, 103]. In order to determine whether CHMP7 and CHMP2B interacted in close spatial proximity within sALS iPSN nuclei, we performed proximity ligation assays (PLA). PLA is a specific and sensitive method enabling the detection of protein – protein interactions in an intact cellular environment. Briefly, hybridized connector oligonucleotides and ligase will join PLA probes (bound to secondary antibodies) to generate a closed DNA circle for amplification when two proteins of interest (detected by specific primary antibodies) are in close proximity to each other. Thus, each discrete PLA spot represents two proteins localized within ~ 40 nm of each other. Using this methodology, we observed a significant increase in the number of CHMP2B – CHMP7 PLA spots in sALS iPSNs at day 25 that was sustained at day 32 compared to control iPSNs (Fig. 6A, C-D). Interestingly, despite the predominant nuclear localization of CHMP7 in sALS iPSNs at day 18 (Supplemental Fig. S12A), we detected few nuclear PLA signals were detected (Fig. 6A-B). This is consistent with the time dependent emergence of pathologic CHMP7/ESCRT-III mediated POM121 reduction in ALS (Figs. 1 and 4 [5, 22, 23]). We also detected an increased number of CHMP2B – CHMP7 PLA spots in postmortem sALS patient motor cortex, but not occipital cortex, which is unaffected in disease, compared to controls (Fig. 6E-G). Consistent with our prior study [20], we did not observe any alteration in CHMP2B subcellular distribution in sALS iPSNs or postmortem patient tissues (Supplemental Fig. S12B). Nonetheless, these data provide evidence for an increased and sustained association between CHMP2B and CHMP7 within sALS patient nuclei in disease. Using in vitro binding assays, we demonstrate that CHMP2B and CHMP7 recombinant proteins have the capacity to directly bind (Supplemental Fig. S13). Although our PLA experiments can not distinguish between directly bound proteins and those in close proximity in multi-protein complexes, our data suggest that CHMP2B and CHMP7 are, at minimum, in close proximity in ALS nuclei (Fig. 6) with the potential to directly bind (Supplemental Fig. S13).

### CHMP2B facilitates the activation of CHMP7/ESCRT-III nucleoporin turnover to initiate pathologic reduction of POM121

Given our observation that CHMP2B interacts with CHMP7 in iPSN nuclei (Fig. 6) and functionally contributes to CHMP7 initiated NPC injury cascades (Figs. 2 and 3, Supplemental Figs. S4–S10) and abnormal turnover of POM121 (Fig. 4) in sALS, we next wanted to investigate the mechanism by which this occurs. We have previously demonstrated that impaired integrity of the passive diffusion permeability barrier of the NPC is a significant contributor to increased nuclear localization of CHMP7 and CHMP7 mediated NPC injury cascades in sALS [5]. To test whether CHMP2B reduction altered passive diffusion through the NPC, we used digitonin permeabilized cell assays [1] to selectively permeabilize the plasma membrane of iPSNs, leaving the nuclear membrane intact, thereby enabling an evaluation of passive influx through the NPC. Similar to our prior publication [5], we observed a significant increase in the fluorescence of a 70 kDa dextran molecule in the nucleus of sALS iPSNs (Supplemental Fig. S14). Interestingly, this was not altered by either siRNA or ASO mediated knockdown of CHMP2B (Supplemental Fig. S14). Due to their size, 70 kDa dextran molecules should normally be unable to passively diffuse through the NPC and are therefore excluded from the nucleus [1, 60, 74, 97] as observed in our control iPSNs (Supplemental Fig. S14). Therefore, these data suggest that knockdown of CHMP2B does not repair NPC permeability barrier integrity and thus, likely acts to alleviate CHMP7 mediated NPC injury via a distinct mechanism.

**Fig. 7:**
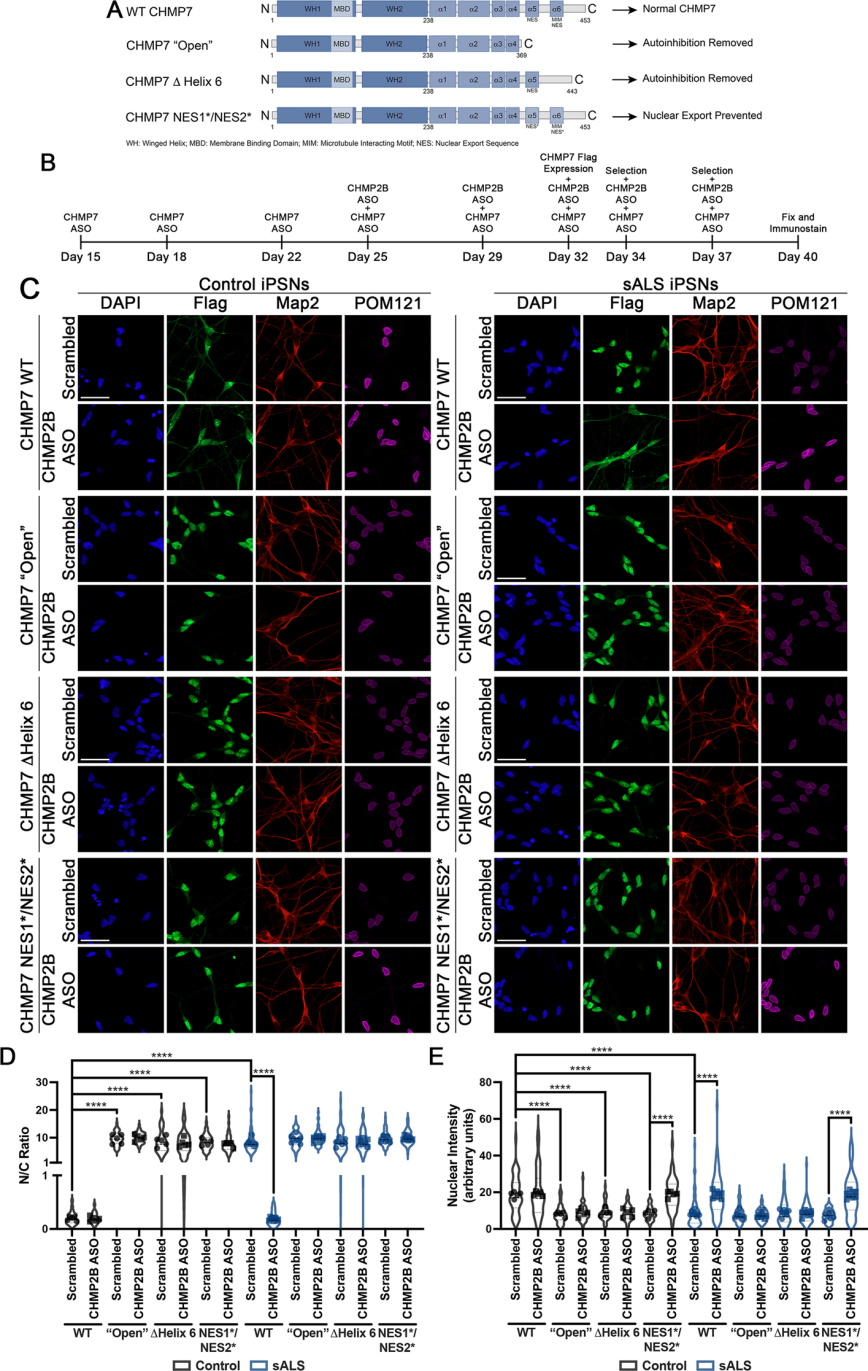
CHMP2B facilitates the activation of CHMP7 to initiate NPC injury in sALS iPSNs. (**A**) Graphical depiction of CHMP7 mutants and their resulting functional consequences. (**B**) Schematic overview of experimental paradigm. (**C**) Maximum intensity projections from immunostaining and confocal imaging of Flag tagged CHMP7 and endogenous POM121 in control and sALS iPSNs. CHMP7 plasmid and ASO treatment as indicated on left, antibody for immunostaining and genotype as indicated on top. (**D**) Quantification of nuclear to cytoplasmic ratios of CHMP7. *n* = 5 control and 8 sALS iPSC lines, 100 Map2 + and Flag + cells per line/overexpression/treatment. Two-way ANOVA with Tukey’s multiple comparison test was used to calculate statistical significance. **** *p* < 0.0001. (**E**) Quantification of POM121 nuclear intensity. *n* = 5 control and 8 sALS iPSC lines, 100 Map2 + and Flag + cells per line/overexpression/treatment. Two-way ANOVA with Tukey’s multiple comparison test was used to calculate statistical significance. **** *p* < 0.0001. Scale bar = 50 μm

ESCRT-III proteins generally reside in an auto-inhibited state to prevent inappropriate polymerization. In order to function properly, they must first be “activated”, a process which involves the removal of auto-inhibition via conformational changes that occur as a result of protein – protein interactions [58, 84, 103]. During nuclear envelope sealing events the inner nuclear membrane protein LEM2/LEMD2 interacts with CHMP7 to ultimately promote its activation in yeast and non-neuronal mammalian cells [36, 68, 95, 105, 107]. In contrast, we demonstrated that LEMD2 is not required for CHMP7 mediated NPC injury events in human iPSNs [5, 23]. Given that previous reports have suggested that interactions amongst individual ESCRT proteins may facilitate their activation [58, 84, 103], we hypothesized that CHMP2B may facilitate CHMP7 activation in iPSNs. To investigate this hypothesis, we first generated a number of Flag tagged CHMP7 mutant plasmid constructs (Fig. 7A). The removal of the C-terminal region of ESCRT-III proteins has previously been shown to eliminate autoinhibition, thereby converting to an “open” conformation to promote activation and polymerization [87]. As a result, similar to studies in yeast [95, 107], we generated a Flag tagged CHMP7 “Open” mutant by removing the last 84 amino acids thereby, truncating CHMP7 at amino acid 369 (Fig. 7A). As an additional method to remove auto-inhibition, we generated a Flag tagged ΔHelix 6 CHMP7 mutant by removing amino acids 420–430 thereby deleting Helix 6 (Fig. 7A). This mutant CHMP7 protein is known to behave similarly to CHMP7 “Open” mutants in promoting the activation of CHMP7 non-neuronal mammalian cells [30]. Notably, both of these approaches also impact the nuclear export sequences (NES) within Helix 5 and Helix 6 of CHMP7 [30]. Thus, by nature of removal of these NES sequences within our “Open” and ΔHelix 6 mutants (Fig. 7A), Flag tagged CHMP7 will be artificially retained within the nucleus as has previously been demonstrated [23, 30, 95, 104]. As we have previously shown that CHMP7 nuclear retention triggers NPC injury in iPSNs [23], we generated a double NES mutant (CHMP7 NES1*/NES2*) by making an L to A substitution at amino acids 388 (NES1) and 430 (NES2) (Fig. 7A) as an additional control to discriminate the impact of activation and that of nuclear accumulation. Previous studies have demonstrated that this double mutation is sufficient to render both NES’ within CHMP7 inactive while maintaining autoinhibitory activity [30, 104, 107]. For comparison, we employed a previously described Flag tagged wildtype CHMP7 plasmid [104] (Fig. 7A). Importantly for our experimental design, as our CHMP7 ASO targets intron 2 of the human CHMP7 pre-mRNA, these plasmids are insensitive to ASO mediated knockdown. To avoid any compounding artifacts that may arise from interactions between endogenous wildtype CHMP7 and our Flag tagged CHMP7 mutants, iPSNs were subjected to treatment with CHMP7 ASOs (Fig. 7B) to deplete endogenous CHMP7, an approach which consistently yields a > 90% reduction in endogenous CHMP7 protein [23, 82]. Flag tagged CHMP7 plasmids were expressed at the neuronal stage (Fig. 7B) to specifically examine NPC maintenance and not NPC formation that would occur during the iPSC or early neuronal differentiation stage. Treatment with neomycin (Fig. 7B) enriched cultures for iPSNs expressing Flag tagged CHMP7 plasmids. Critically, immunostaining and confocal imaging for Flag demonstrated that ASO mediated reduction of CHMP2B mitigated the nuclear accumulation of Flag tagged WT CHMP7 in sALS iPSNs (Fig. 7C-E) similar to our results for endogenous CHMP7 (Fig. 2). In addition, all three CHMP7 mutants localized to the nucleus as expected (Fig. 7C-E). This highlights out approach to “replace” endogenous CHMP7 with Flag tagged CHMP7 variants as a viable strategy for investigating the mechanisms by which ESCRT-III proteins interact to facilitate NPC injury events in disease iPSNs.

Having validated our experimental approach, we next performed immunostaining and confocal imaging to evaluate the role of CHMP2B in CHMP7 mediated NPC injury, namely the reduction of nuclear POM121 immunoreactivity. As expected, we observed a significant reduction in POM121 immunoreactivity in sALS iPSNs expressing Flag tagged WT CHMP7 that was abrogated by treatment with CHMP2B ASOs (Fig. 7C-E). We next tested whether CHMP2B was involved in the activation of CHMP7 by utilizing our Flag tagged CHMP7 “Open” and ΔHelix 6 plasmids in which auto-inhibition capabilities have been removed (Fig. 7A), thereby artificially “activating” CHMP7 as established by previous studies [30, 87, 107]. Expression of either Flag tagged CHMP7 “Open” or Flag tagged CHMP7 ΔHelix 6, resulted in a significant decrease in nuclear POM121 immunoreactivity in control and sALS iPSNs (Fig. 7C-E). However, in striking contrast to our results obtained in the context of endogenous (Fig. 3) or Flag tagged WT CHMP7 (Fig. 7C-E) expression, ASO mediated knockdown of CHMP2B had no impact on POM121 immunoreactivity in iPSNs expressing Flag tagged CHMP7 “Open” or Flag tagged CHMP7 ΔHelix 6 (Fig. 7C-E). Simply retaining CHMP7 in the nucleus by abrogating it’s nuclear export (CHMP7 NES1*/NES2*) resulted in a significant decrease in POM121 immunoreactivity which was mitigated by ASO medicated reduction of CHMP2B (Fig. 7C-E). This suggests that in the absence of CHMP2B, CHMP7 nuclear retention alone is not sufficient to initiate POM121 reduction in iPSNs. Critically, PLA confirmed that CHMP7 “Open” and CHMP7 ΔHelix 6 no longer associated in close spatial proximity to CHMP2B in iPSN nuclei (Supplemental Fig. S15). Therefore, these data collectively support a role for CHMP2B in facilitating and sustaining the activation and/or removal of autoinhibition of CHMP7 to initiate NPC injury in sALS iPSNs.

## Discussion

**Fig. 8:**
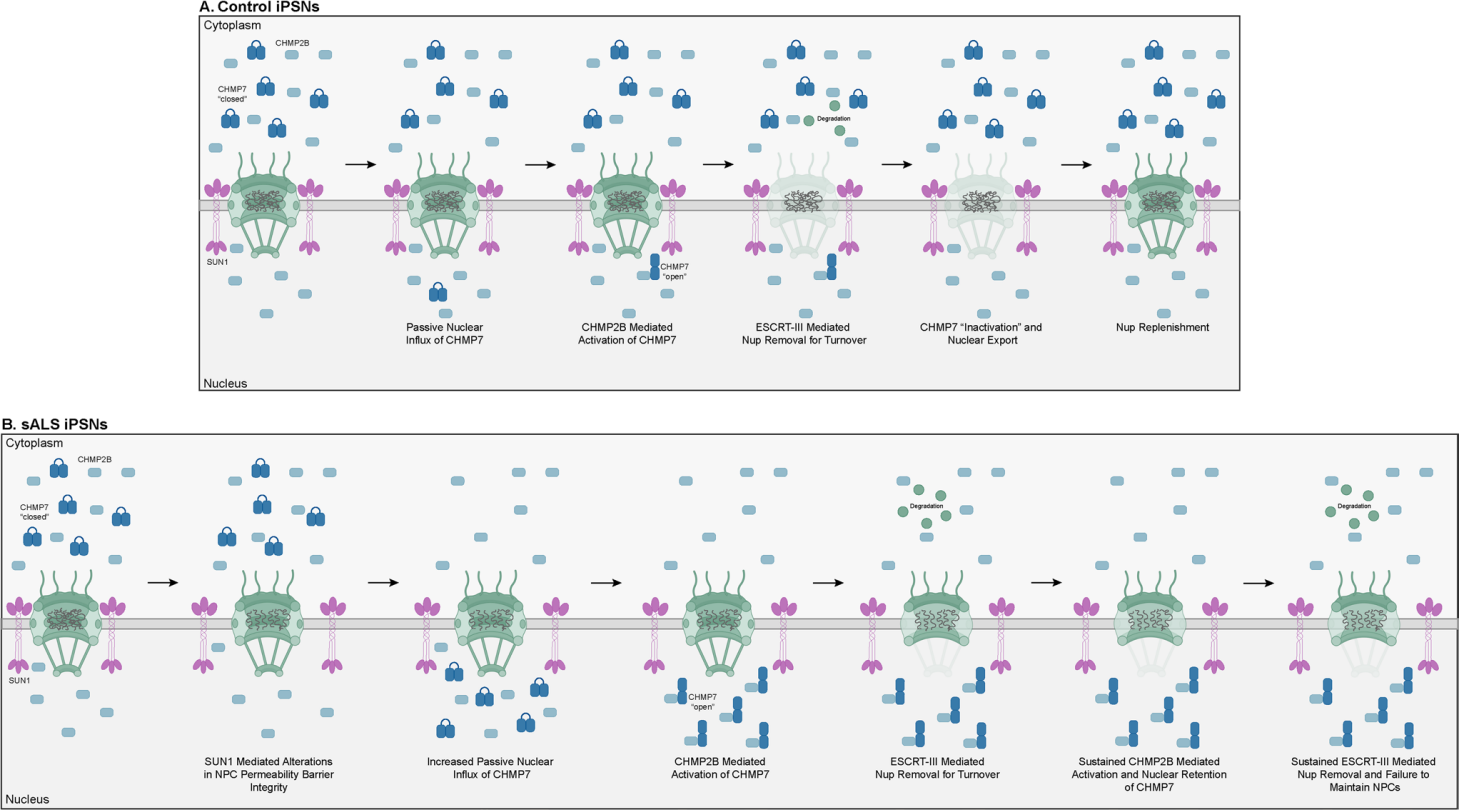
Graphical model depicting role of CHMP2B in facilitating CHMP7 nuclear retention and activation for pathologic ESCRT-III mediated Nup degradation in sALS. (**A**) In control human neurons for normal NPC maintenance and Nup turnover, the ESCRT-III protein passively diffuses through the NPC where it undergoes activation via association with CHMP2B. Following ESCRT-III mediated Nup degradation, CHMP7 is inactivated and actively exported from the nucleus. Nup molecules are replenished, and NPC homeostasis is maintained. (**B**) In sALS neurons, SUN1 mediated disruptions in NPC permeability barrier integrity [5] lead to increased passive diffusion of CHMP7 into the nucleus where it undergoes activation via association with CHMP2B leading to degradation of specific Nups from the NPC. CHMP2B mediated activation of CHMP7 is sustained leading to continued ESCRT-III mediated Nup removal and degradation giving rise to NPC pathology observed in sALS

Impaired nucleocytoplasmic compartmentalization has emerged as significant contributor to ALS and related neurodegenerative disease pathophysiology. We have recently demonstrated that increased nuclear influx of the ESCRT-III protein CHMP7 resulting from SUN1 mediated alterations in NPC permeability barrier integrity occurs prior to documented reduction of specific Nups from the NPC itself in sALS iPSNs [5, 23]. However, the molecular mechanisms by which increased nuclear CHMP7 localization impacts pathologic alterations to the repertoire of Nups within the nucleus and NPCs of sALS neurons remains unknown. In this study, we demonstrate a role for the ESCRT-III protein CHMP2B in the activation of physiologic and pathologic CHMP7/ESCRT-III Nup turnover in human neurons. In aggregate, our data presented in this manuscript suggests a model whereby prolonged CHMP2B mediated overactivation of CHMP7/ESCRT-III Nup turnover leads to pathologic NPC injury in sALS iPSNs (Fig. 8). Importantly, CHMPB mediated activation of the CHMP7/ESCRT-III nuclear surveillance pathway appears to facilitate CHMP7’s nuclear localization in sALS neurons highlighting the importance of ESCRT-III function in the determination of subunit localization. In turn, we demonstrate two approaches to reduce CHMP2B protein expression, namely siRNA and ASO mediated knockdown, that abrogate sustained “over-activation” of Nup turnover thereby alleviating pathophysiologic events associated with NPC injury including POM121 expression and TDP-43 dysfunction.

A number of reports have now established a role for CHMP7 in nuclear envelope resealing during cell division [36, 68, 95, 105–107]. In addition, one study has suggested that ESCRT-III proteins including CHMP2B and CHMP7 can promote the maintenance of nuclear/cytoplasmic compartmentalization during times of nuclear envelope rupture in yeast [2]. Although nuclear envelope abnormalities have been reported in models of C9orf72 ALS/FTD and Profilin1 ALS [34, 88], it remains unclear whether these represent reported alterations to resident nuclear envelope proteins [5, 89] or pathologic rupture sites. Thus, future studies are necessary to determine whether small ruptures within the nuclear envelope impact ESCRT-III nuclear surveillance in ALS. Nonetheless, we have recently demonstrated that abnormal nuclear localization of CHMP7 is sufficient to initiate pathologic alterations to the NPC itself [23] thereby highlighting a pivotal role for CHMP7 and the ESCRT-III nuclear surveillance pathway in NPC disruption in sALS. However, the mechanisms by which this pathologic increase in nuclear localization of CHMP7 drives disease associated changes to the NPC remains unknown.

In addition to a role in nuclear envelope sealing, recent studies have also implicated CHMP7 and the ESCRT-III pathway in the proper assembly and insertion of NPCs into the nuclear envelope during cell division [30, 36, 64, 95, 105–107]. However, little is known regarding the function of ESCRT-III proteins in the maintenance of previously assembled NPCs throughout the lifetime of non-dividing cells such as neurons where entire NPCs are not thought to be reassembled and inserted throughout life. In fact, a number of Nups have been reported to be some of longest lived proteins in the mammalian CNS [98]. As a result, in order to maintain properly functioning NPCs, it is possible that individual Nup protein molecules are turned over and replenished within NPCs throughout the lifetime of neurons. Indeed, this is supported by a prior study suggesting that Nups are exchanged at varying rates in myoblast and myotube nuclei resulting in individual NPCs containing a mix of new and old Nup proteins [99]. We now provide evidence supporting a fundamental role for the ESCRT-III pathway and two of its protein constituents, CHMP7 and CHMP2B, in piecemeal POM121 Nup turnover in human iPSNs (Figs. 1 and 4). Specifically, we demonstrate that reduction of either CHMP7 or CHMP2B significantly slows POM121 turnover in control human iPSNs (Figs. 1 and 4). In addition, it appears this fundamental function of the ESCRT-III pathway is “overactive”, hastening POM121 turnover in sALS iPSNs (Figs. 1 and 4) thereby giving rise to NPC pathology observed in disease [22, 23], a process once again slowed by reduction of either CHMP7 or CHMP2B (Figs. 1 and 4). In the future, as imaging technologies advance, it will be interesting to determine how many molecules of POM121, a Nup central to NPC injury cascades [22], are exchanged in each NPC and with what frequency within the 1–2 week time frame examined in this current study. This will be particularly important when technically feasible given our previous documentation of the heterogeneity of POM121 reduction (e.g. number of spots, intensity of spots) to initiate compositional and functional changes in the NPC in ALS iPSNs [22]. Nonetheless, our data is consistent with prior reports documenting a role for ESCRT-III proteins CHMP3 and CHMP2A in piecemeal turnover of Nup93 in non-dividing mouse myoblast cells [99]. Collectively, this highlights an essential role for the nuclear surveillance function of the ESCRT-III pathway in maintenance of NPCs throughout the lifetime of non-dividing cells.

To maintain NPC and NE homeostasis, the ESCRT-III protein CHMP7 passively diffuses into the nucleus, typically in its inactive autoinhibited or “closed” state which prevents aberrant ESCRT polymerization and unregulated function. In order for ESCRT-III mediated NPC and nuclear envelope surveillance and maintenance events to occur, CHMP7 must then transition into an active or “open” state via protein – protein interactions. Typically, for ESCRT-III mediated nuclear surveillance and maintenance events in cell division, CHMP7 interacts with the inner nuclear membrane protein LEMD2 to facilitate its “activation” and polymerization [36, 68, 95, 105, 107]. In addition, prior reports have demonstrated that interactions between individual ESCRT proteins can result in their activation [58, 84, 103] highlighting the existence of multiple routes to ESCRT protein activation. Given our previous publications demonstrating that LEMD2 is not required for CHMP7’s nuclear localization and function in iPSNs [5, 23], we reasoned that specific ESCRT protein interactions may facilitate the nuclear localization and activation of CHMP7/ESCRT-III function in iPSNs. Indeed, upon reduction of CHMP2B, CHMP7 relocalized to the cytoplasm in sALS iPSNs (Fig. 2) although passive diffusion through the NPC remained dysregulated (Supplemental Fig. S14). By utilizing CHMP7 mutant plasmids in which autoinhibition has been removed, thereby promoting constitutive activation of CHMP7, we demonstrate that CHMP2B is no longer required for pathologic POM121 reduction (Fig. 7). Thus, together with our observations that knockdown of CHMP2B mitigates POM121 reduction (Fig. 3) and slows POM121 turnover (Fig. 4) in sALS iPSNs, these data suggest that CHMP2B is sufficient to facilitate the activation of the CHMP7/ESCRT-III NPC surveillance pathway in iPSNs and is a critical mediator of ESCRT-III nuclear surveillance overactivation in sALS. In aggregate, our study establishes not only that CHMP2B is required for pathologic ESCRT-III NPC alterations, but that functionality of ESCRT-III proteins, in this case CHMP7, is an important contributor to their localization in human neurons. This is consistent with prior ESCRT-III pathway studies suggesting that ESCRT-III function proceeds in a stepwise sequence involving localization/membrane targeting, removal of autoinhibition, polymerization of ESCRT-III subunits, VPS4 mediated scission and depolymerization following completion of remodeling events, restoration of autoinhibition, and redistribution throughout the cell [41, 42, 46, 58, 103]. Thus, although many factors including NPC permeability barrier integrity and RNA splicing can impact localization of CHMP7 [3, 5], our study highlights the importance of ESCRT-III protein function in the determination of their localization.

Using proximity ligation, we observed an increased and sustained association between CHMP2B and CHMP7 in sALS iPSNs and postmortem human tissues (Fig. 6). We hypothesize that under physiologic conditions, nuclear pools of CHMP7 and CHMP2B interact in close proximity in order to facilitate physiologic ESCRT-III mediated Nup turnover. Indeed, we demonstrate that impaired association between CHMP7 and CHMP2B via ASO mediated reduction of either ESCRT-III subunit dramatically slows both the physiologic and pathologic turnover of POM121 within NPCs in human neurons (Figs. 1 and 4). Although CHMP2B nuclear localization is minimal in control and sALS human neurons (Supplemental Fig. S12), it is likely that increased nuclear localization of CHMP7 observed in sALS leads to increased detection of close physical proximity to CHMP2B in ALS neurons (Fig. 6). Although PLA can not distinguish between direct binding and proximity within protein complexes, in vitro binding experiments suggest that CHMP7 and CHMP2B proteins can directly bind (Supplemental Fig. S13). The stoichiometry of this relationship and whether this direct binding is enhanced by the presence of additional CHMP7 protein will be an important topic of future investigation.

Taken together with our recent publication [5], we propose a scenario whereby sALS associated CHMP7 pathology is at least in part the result of increased passive nuclear influx resulting from SUN1 mediated alterations in NPC permeability barrier integrity [5] combined with sustained CHMP2B mediated activation of CHMP7/ESCRT-III nuclear surveillance within the nucleus (Fig. 8), highlighting a role for function in determining ESCRT-III protein localization in human neurons. Interestingly, ASO mediated reduction of CHMP2B is still sufficient to mitigate the reduction of POM121 that results from artificial nuclear retention of CHMP7 via impaired nuclear export (CHMP7 NES1*/NES2*, Fig. 7). Although future investigation is warranted, this data demonstrates that in sALS neurons, impaired nuclear export of CHMP7 alone may not be a primary driver of CHMP7 mediated NPC injury in disease. Further, we propose a scenario whereby persistent interactions between CHMP7 and CHMP2B leads to over-activation/prolonged activation of ESCRT-III mediated Nup removal and degradation to give rise to NPC pathology observed in sALS (Fig. 8). However, it remains unclear at this time whether this sustained interaction is the result of continued nuclear influx of CHMP7 thus providing a constant flow of new CHMP7 molecules for activation, and/or through an inability to dissociate activated ESCRT-III protein polymers following their NPC and surveillance function. Typically, recruitment of the AAA-ATPase VPS4 promotes ESCRT-III polymer disassembly [12, 24, 38, 56, 58, 84, 103, 110]. We have recently demonstrated that VPS4 is recruited and pathologically increased in a CHMP7 dependent manner in ALS nuclei [20]. However, CHMP7 is unable to directly bind to VPS4 due to a charge substitution within its Microtubule Interaction and Trafficking Domain Interaction Motif (MIM) domain in Helix 6 [30]. Thus, it is likely that other ESCRT-III protein subunits are involved facilitating recruitment of VPS4 for polymer disassembly after ESCRT-III nuclear surveillance function. Interestingly, prior studies indicate that CHMP2B is able to bind to VPS4 in vitro [93, 108]. However, given our data that reduction of CHMP2B alleviates pathologic reduction of POM121 (Figs. 3 and 4), this suggests that CHMP2B is unlikely involved in recruitment of VPS4 for nuclear ESCRT-III polymer disassembly in human neurons. Thus, future investigations are necessary to examine the function of additional ESCRT-III subunits in promoting VPS4 recruitment and polymer disassembly in iPSNs. Nonetheless, taken together, we demonstrate that the fundamental ESCRT-III nuclear surveillance pathway is involved in piecemeal turnover of POM121 in human neurons. Critically, given that aberrant POM121 reduction is the initiating event for NPC injury cascades in ALS neurons [22], our study provides essential mechanistic insights into this early event in ALS pathogenesis.

Notably, mutations in CHMP2B have been implicated in FTD and a subset of lower motor neuron predominant ALS cases [19, 66, 91, 100]. Mutations resulting in a C terminal truncation, thereby removing CHMP2B’s autoinhibitory domain and VPS4 binding site, have been shown to enhance the association between CHMP2B and CHMP4B [49] and impair the recruitment of VPS4 [18]. This suggests that disease associated mutations in CHMP2B may additionally lead to NPC injury events in this genetic form of FTD/ALS in a manner similar to our observations in sALS. However, the impact of disease associated mutations in CHMP2B on ESCRT-III nuclear surveillance and NPC homeostasis remains unclear at this time and is a topic of investigation we hope to pursue in the of future.

Collectively, our data provides evidence of a role for CHMP2B in facilitating the activation of the CHMP7/ESCRT-III nuclear surveillance pathway in human iPSNs to maintain NPCs. Moreover, we demonstrate that sustained activation of CHMP7/ESCRT-III nuclear surveillance is sufficient to drive NPC injury in sALS. Consequently, ASO or siRNA mediated knockdown of CHMP2B mitigates pathologic reduction of POM121 and downstream defects in TDP-43 function in sALS iPSNs. As a result, our data mechanistically implicate CHMP2B mediated activation of CHMP7/ESCRT-III nuclear surveillance as a significant contributor to physiologic NPC maintenance and pathologic NPC disruption in human neurons. Prior studies have reported deficiencies in neurite extension and branching as well as synaptic dysfunction in cultured rodent neurons following shRNA mediated depletion of CHMP2B [14] consistent with a role for CHMP2B at the synapse [14, 17]. However, consistent with the observation that CHMP2B knockout mice are largely phenotypically normal [33], our study suggests that only a partial reduction of CHMP2B following neuronal development may be beneficial in ALS. In support of this, recent observations indicate that knockdown of CHMP2B prevents toxicity resulting from TDP-43 overexpression in *Drosophila* and can modulate the phosphorylation of TDP-43 in overexpression models [27]. Thus, in addition to providing critical mechanistic insights into disruptions in CHMP7/ESCRT-III mediated NPC homeostasis, our data support the potential for partial reduction of CHMP2B as a potential therapeutic strategy to alleviate NPC injury cascades in sALS. Although future studies are necessary to extend these studies to a larger cohort of sALS patient iPSC lines, our recent analysis of 180 control, sALS, C9orf72, TDP-43, and SOD1 iPSC lines has revealed that ~ 75% of patient lines display CHMP7 pathology and ~ 85% have evidence of aberrant POM121 reduction [82]. Thus, this suggests that therapeutically targeting overactivation of ESCRT-III mediated POM121 turnover may be beneficial for a large number of sALS patients. Given the potential for direct binding between CHMP7 and CHMP2B (Supplemental Fig. S13), our study also suggests the possibility that small molecule inhibitors of sustained nuclear CHMP7 – CHMP2B interactions may be a future alternative therapeutic approach to alleviate NPC injury cascades in disease.

## Conclusions

In this study, we demonstrate that CHMP2B is required for physiologic neuronal Nup turnover and in turn is a significant mediator of pathologic NPC injury in sALS human neurons. Mechanistically, CHMP2B associates with CHMP7 to promote the activation of ESCRT-III mediated Nup turnover. In sALS neurons, the persistent association between CHMP7 and CHMP2B leads to sustained overactivation of the ESCRT-III nuclear surveillance/Nup turnover pathway to give rise to disease associated NPC injury and in turn contribute to TDP-43 mislocalization and loss of nuclear function. Importantly, we demonstrate that partial reduction of CHMP2B via siRNA or ASO is sufficient to both prevent and reverse NPC alterations and TDP-43 pathology. Thus, these studies provide critical mechanistic insights into early events in sALS pathogenesis and highlight CHMP2B as a potential therapeutic target for sALS.

## Methods

### iPSC maintenance and differentiation

Control and sALS iPSC lines were obtained from the Answer ALS [6] repository at Cedars Sinai (see Supplemental Table S1 for demographics information). Based on our recent analysis of 180 iPSC lines [82], we selected patient iPSC lines with similar patterns of molecular hallmarks of TDP-43 loss of function were selected for this study. iPSCs were maintained in mTeSR Plus media as previously described [5]. Mixed spinal neuron cultures were generated using a recently described modified direct induced motor neuron (diMNs) differentiation protocol [5]. All iPSC and iPSN cultures were maintained at 37 °C with 5% CO_2_ and routinely tested negative for mycoplasma.

### ASO Treatment of iPSNs

Previously described non-targeting scrambled control and human CHMP7 targeting ASOs [23] were generously provided by Ionis Pharmaceuticals. Human CHMP2B targeting ASOs were independently designed and synthesized by IDT. All ASOs are gapmers and contain phosphorothioate bonds and are 2’-O-mthoxyethyl (2’MOE) modified. ASO sequences are provided in Supplemental Table S2. CHMP2B ASO 3 was used for all iPSN experiments throughout this manuscript. ASO dosing (5 µM) was initiated at the time points indicated in figure legends. Media was exchanged and ASO replenished every 3–4 days for the duration of the experiment.

### siRNA mediated Knockdown in iPSNs

Dharmacon ON-TARGETplus SMARTPool CHMP2B and non-targeting (NT) SMARTPool siRNAs (Horizon Discovery) were delivered to iPSNs using the Lonza 4D nucleofection system. At time point indicated in figure legends, iPSNs were nucleofected with 500 nM siRNA in suspension using the Lonza P3 Primary Cell 4D Nucleofector Kit and program DC-104. Following nucleofection, iPSNs were replated and media was exchanged the following day and every 3–4 days thereafter for the duration of the experiment and as indicated in figure legends.

### Plasmid expression in iPSNs

CHMP7 and POM121 RITE plasmids and sources are detailed in Supplemental Table S3. Flag tagged CHMP7 Open, CHMP7 ΔHelix 6, and CHMP7 NES1*/NES2* plasmids were synthesized by Genscript. Gene sequences were cloned into the pcDNA3.1 plasmid backbone using the BamHI and XhoI restriction sites. CHMP7 Open was generated by removing the last 84 amino acids, truncating CHMP7 at amino acid 369 [30, 95]. CHMP7 ΔHelix 6 was generated by removing amino acids 420–430 [95]. CHMP7 NES1*/NES2* was generated by making L to A amino acid substitutions at amino acids 388 and 430 within the CHMP7 NES1 and NES2 sequences respectively [30, 95]. At the time points indicated in figure legends, iPSNs were dissociated with accutase to generate a single cell suspension as previously described [5, 22, 23]. Plasmid DNA was delivered to iPSNs in suspension using the P3 Primary Cell 4D Nucleofector Kit and program DC-104 on the 4D Nucleofector (Lonza). Cuvettes contained 4 × 10^6 iPSNs and 4 µg plasmid DNA. Following nucleofection, iPSNs were replated. Media was exchanged 24 h later and every 3–4 days thereafter for the duration of the experiment. For our experiments utilizing Flag tagged CHMP7 plasmids, at every media change, iPSNs were treated with neomycin for 24 h to enrich for iPSNs expressing plasmid DNA of interest.

### Immunostaining and confocal imaging in iPSNs

To facilitate monolayer based imaging, iPSNs were replated in glass bottom imaging plate (Cellvis) 10 days prior to fixation In accordance with our recently described protocol [5]. At time points indicated in figure legends, iPSNs were fixed with 4% paraformaldehyde (PFA) in 1X PBS for 15 min at room temperature. iPSNs were subsequently washed 3 × 10 min with 1X PBS at room temperature and permeabilized with 1X PBS containing 0.01% Triton X-100 for 15 min at room temperature. Blocking was then carried out by incubating iPSNs in a 10% goat serum/1X PBS solution for 30 min at room temperature. iPSNs were then incubated with primary antibodies (see Supplemental Table S4) diluted in block solution for 2 h at room temperature. Following incubation with primary antibodies, iPSNs were washed 3 × 10 min with 1X PBS and then incubated with secondary antibodies (see Supplemental Table S4) diluted in block solution for 45 min at room temperature. iPSNs were then washed 1X with 1X PBS, incubated with Hoechst solution (1:1000 in 1X PBS) for 10 min at room temperature, and finally washed with 1X PBS for 10 min. Rotation was not used at any step. Addition and removal of all solutions was conducted manually with a P1000 pipette to prevent lifting of iPSNs from the dish. iPSNs were mounted using Prolong Gold Antifade Reagent and sandwiched with a 15 mm glass coverslip to facilitate imaging and storage. Confocal imaging was carried out using a 20X or 63X objective on a Zeiss LSM 980 with AiryScan 2 confocal microscope. Standard frame scanning confocal imaging parameters were used for image acquisition. All images were acquired using identical imaging parameters for each immunostained protein within a given experiment. Nuclear, cytoplasmic, and whole cell intensities were measured in FIJI. Regions of interest were manually outlined using DAPI and Map2 to define nuclear and cell body compartments respectively as has been previously described [5, 22, 23]. Integrated density was then measured in the channel of interest based on defined regions of interest. Corrected total cell fluorescence (CTCF) measurements for the nucleus and cell body were calculated as follows: CTCF = integrated density – (area of region of interest x mean fluorescence background reading). Cytoplasmic CTCFs were calculated by subtracting nuclear CTCF from cell body CTCF. Nuclear/cytoplasmic ratios were calculated as previously described [5, 22, 23]. Genotypes and treatment were blinded for imaging and analysis. All images are presented as maximum intensity projections generated with Zeiss Zen Blue 2.3 software.

### RITE and AiryScan imaging

POM121 RITE plasmids were expressed in iPSNs as described above. To allow for full incorporation into the NPC, tag exchange was induced one week following nucleofection by adding 4-hydroxytamoxifen (4-OHT) directly to media to a final concentration of 1 µM. Media was exchanged and 4-OHT replenished every 3–4 days for 1–2 weeks. At time points indicated in figure legends, iPSNs were fixed and immunostained as detailed above. AiryScan imaging was carried out using the AiryScan 2 super resolution module on a Zeiss LSM 980 confocal microscope. All images were acquired with a 63X objective and 3X digital zoom. Identical imaging parameters (e.g. laser power, gain) were used to acquire all images. The number of Myc and Flag tagged POM121 spots were calculated using automated analysis pipelines where regions of interest are defined by DAPI masks in FIJI that have been previously described [5, 22, 23]. Double counting was avoiding by analyzing each tag individually and then analyzing a colocalized image. The number of colocalized Myc/Flag spots was subtracted from the total of each individual count. Percentages of “old” (Myc), “new” (Flag), or “mixed” (Myc/Flag) POM121 spots were calculated by dividing by the total number of spots detected. Genotypes, treatment, and time points were blinded for imaging and analysis.

### Immunofluorescent staining and imaging of postmortem human tissue

Formalin fixed paraffin embedded postmortem motor and occipital cortex tissue slides were obtained from the Target ALS Human Postmortem Tissue Core. Demographic information for non-neurological control and sALS patient tissues can be found in Supplemental Table S5. Deparaffinization was carried out by immersing slides 3 × 5 min in xylene followed by gradual rehydration of tissue sections by immersing in a series of washes for 5 min each: 2 × 100% ethanol, 1 × 90% ethanol, 1 × 70% ethanol, 3X dH_2_O. Antigen retrieval was carried out by incubating slides in Tissue-Tek antigen retrieval solution (IHC world) in a steamer for 1 h. Following 10 min cooling at room temperature, slides were washed 3 × 5 min with dH_2_O and then 2 × 5 min with 1X PBS. Tissue sections were permeabilized using a 0.4% Triton-X solution (in 1X PBS) for 10 min on a shaker and then washed 3 × 5 min with 1X PBS. Slides were blocked overnight at 4 °C in DAKO protein-free serum block (DAKO) and then incubated with primary antibody (see Supplemental Table S4 for antibody information) diluted in DAKO antibody diluent reagent with background reducing agents (DAKO) for 48 h at 4 °C. Tissue sections were then washed 3 × 5 min with 1X PBS and incubated with secondary antibody (see Supplemental Table S4 for antibody information) diluted in DAKO antibody diluent reagent with background reducing agents (DAKO) for 1 h at room temperature. Slides were washed 3 × 5 min with 1X PBS, incubated with 2–3 drops of autofluorescence eliminator reagent (Millipore) for 10 s and washed 5 × 5 min with 1X PBS. Tissue sections were then incubated with Hoechst solution (1:1000 in 1X PBS) for 20 min and then subsequently washed 3 × 5 min with 1X PBS. The final PBS wash was exchanged for dH_2_O and then slides were cover slipped using Prolong Gold Antifade Reagent. Imaging of Map2 positive Layer V neurons was carried out with a 20X objective on a Zeiss Axioimager Z2 fluorescent microscope with an Apotome2. Identical imaging parameters (e.g. exposure time) were used for all images and 10 images were acquired per slide. Zeiss Zen Blue 2.3 was used to generate default apotome processed images.

### Proximity ligation assay (PLA)

iPSNs: At time points indicated in figure and legends, iPSNs were rinsed 2X with 1X PBS and fixed in 4% PFA (in 1X PBS) for 15 min. Fixed iPSNs were then washed 3 × 5 min with 1X PBS and permeabilized with 0.01% PBST (1X PBS containing 0.01% Triton X-100) for 15 min at room temperature. Proximity ligation was then carried out in accordance with the Duolink PLA Fluorescence protocol (Sigma Aldrich). Briefly, iPSNs were blocked with Duolink Blocking solution in a humidified 37 °C incubator for 1 h and then incubated with primary antibodies (CHMP2B and CHMP7, see Supplemental Table S4) diluted in 1X Duolink Antibody Diluent for 2 h at room temperature. iPSNs were washed 2 × 5 min with 1X Wash Buffer A at room temperature and then incubated with Duolink Anti-Rabbit PLUS and Duolink Anti-Mouse MINUS probes in 1X Duolink Antibody Diluent (80 µL total solution per well of a Cellvis 24 well glass bottom plate) in a humidified 37 °C incubator for 1 h. Following probe incubation, iPSNs were washed 2 × 5 min with 1X Wash Buffer A at room temperature and ligation was then carried out by incubation with Ligase diluted 1:40 in 1X Ligation buffer (80 µL total solution per well of a Cellvis 24 well glass bottom plate) in a humidified 37 °C incubator for 30 min. iPSNs were washed 2 × 5 min with 1X Wash Buffer A at room temperature and amplification was performed by incubating with Polymerase diluted 1:80 in 1X Amplification buffer (80 µL total solution per well of a Cellvis 24 well glass bottom plate) in the dark in a humidified 37 °C incubator for 100 min. Following amplification, iPSNs were washed 2 × 10 min with 1X Wash Buffer B and 1 × 1 min with 0.01X Wash Buffer B at room temperature. One to two drops of Duolink In Situ Mounting Medium with DAPI was added to each well and coverslips were used to seal each well of the plate. All buffers were made fresh prior to use. Confocal imaging was performed using a 63X objective on a Zeiss LSM 980 with AiryScan 2 confocal microscope. Identical imaging parameters (e.g. laser power, gain) were used for all samples. Individual PLA signals within nuclei were counted using automated spot detection in regions of interest defined by DAPI masks on maximum intensity projections in FIJI. Genotypes and time point were blinded for imaging and analysis.

Postmortem Human Tissue: Postmortem human tissue slides were subjected to deparaffinization, antigen retrieval, washing, and permeabilization as detailed above. Tissue sections were blocked with Duolink Blocking solution for 1 h at 37 °C in a humidified chamber and incubated with primary antibody (CHMP7 and CHMP2B; see Supplemental Table S4) diluted in Duolink Antibody Diluent for 48 h at 4 °C. Proximity ligation was then carried out as described above for iPSNs. Following the final wash with 0.01X Wash Buffer B, slides were incubated with autofluorescence eliminator reagent for 10 s and extensively washed with 1X PBS (5 × 5 min) prior to cover slipping with Duolink In Situ Mounting Medium with DAPI. Cover slips were sealed with nail polish and tissue sections imaged using a 63X objective on a Zeiss Axioimager Z2 fluorescent microscope with an Apotome2. Layer V was identified based on DAPI and 10–20 images were obtained for each slide. Identical imaging parameters (e.g. exposure time) were used for all images and slides. Zeiss Zen Blue 2.3 was used to generate default apotome processed images used for analysis. Individual PLA signals within nuclei were counted using automated spot detection in regions of interest defined by DAPI masks in FIJI. Genotypes and brain region were blinded for imaging and analysis.

### Digitonin permeabilized iPSN assays

Digitonin permeabilized iPSN assays were performed as recently described [5]. iPSNs were first rinsed with 1X PBS and then permeabilized in permeabilization buffer (10% OptiPrep, 200 µg/mL digitonin) on ice for 5 min. iPSNs were then rinsed briefly with ice cold transport buffer (4 mM HEPES-KOH pH 7.5, 22 mM KOAc, 0.4 mM Mg(OAc)_2_, 1 mM NaOAc, 0.1 mM EGTA, 50 mM sucrose) and then washed 3 × 5 min with ice cold transport buffer. Permeabilized and washed iPSNs were then incubated with 0.6 mg/mL fluorescent 70 kDa dextran at room temperature for 25 min protected from light. One drop of NucBlue Live Ready Probes (Thermo Fisher Scientific) was added to each well during incubation. iPSNs were imaged immediately following incubation with a Zeiss LSM 930 with AiryScan 2 confocal microscope. A 40X objective and standard line scanning confocal parameters were used to acquire single z section images. Plates were discarded after 15 min. FIJI was used to determine the nuclear intensity of fluorescent 70 kDa dextran. Genotypes and treatment were blinded for imaging and analysis.

### Western blot

To generate iPSN lysates, at time points indicated in figure legends, iPSNs were rinsed with pre-chilled 1X PBS. RIPA buffer (Sigma Aldrich) supplemented with 1X EDTA free protease inhibitor cocktail (Roche) was added directly to the wells of the culture plate and iPSNs were incubated on ice for 5 min. Lysates were then collected by scraping with a cell scraper, transferred to an Eppendorf tube, and vortexed vigorously for 30 s to facilitate complete iPSN lysis. Lysates were cleared of debris via centrifugation at 12,000 g for 15 min at 4 °C. Supernatants were transferred to a new Eppendorf tube and protein concentrations were determined using a BCA protein estimation kit (Thermo Fisher Scientific). 4X Laemmli buffer (BioRad) supplemented with β-mercaptoethanol was added to each sample to a final concentration of 1X. To obtain nuclear lysates, nuclei were first isolated from iPSNs using the Nuclei PURE Prep Nuclei Isolation Kit (Sigma Aldrich) as previously described [22]. Isolated nuclei were then lysed and lysates prepared as described above. Immediately prior to SDS-PAGE, sampled were heated at 100 °C for 5 min and cooled to room temperature. 10 µg of protein was loaded in 4–20% acrylamide gels (BioRad). Gels were run until the dye front ran off and protein was transferred onto a nitrocellulose membrane with the Trans-Blot Turbo Transfer System (BioRad). Membranes were blocked with 5% nonfat milk (BioRad) in 1X TBST (1X TBS with 0.1% Tween-20) for 30 min on a shaker at room temperature. Blots were incubated overnight on a shaker at 4 °C with primary antibody diluted in block solution (see Supplemental Table S4 for antibody information). Following primary antibody incubation, blots were washed 3 × 10 min with 1X TBST and incubated with secondary antibody diluted in block (see Supplemental Table S4 for antibody information) on a shaker for 1 h at room temperature. Blots were then washed 3 × 10 min with 1X TBST. ECL substrate (Thermo Fisher Scientific, Millipore) was applied for 30 s, and chemiluminescent images were acquired with the ImageQuant LAS 4000 system (GE Healthcare). To allow for sequential probing of blots without stripping [86], chemiluminescent signals were quenched by incubating membranes in 30% H_2_O_2_ for 15 min on a shaker at room temperature. Analysis of signal density was carried out in FIJI. GAPDH was used as a loading control for normalization.

### qRT-PCR

At time points indicated in figure legends, iPSNs were incubated with 1 mL Trizol in wells of the culture plate for 5 min at room temperature. Solutions were then transferred to an Eppendorf tube and Trizol based RNA isolation then proceeded in accordance with manufacturer protocol (Invitrogen). cDNA synthesis was carried out using the High Capacity cDNA Reverse Transcription Kit (Thermo Fisher Scientific). 1 µg of RNA was used for each reaction. All qRT-qPCR reactions were conducted using SYBR Green Master Mix or TaqMan Gene Expression Master Mix (Thermo Fisher) and an Applied Biosystems QuantStudio 3 (Applied Biosystems). Previously described primer sets (see Supplemental Table S6) [55, 59, 78, 85] were used to detect truncated STMN2 and cryptic exon containing mRNA transcripts. TaqMan Gene Expression Assays (see Supplemental Table S6) were used to detect mRNA targets. GAPDH was used for normalization of gene expression.

### In vitro binding

For each reaction, 2 µg CHMP7 His (Cusabio), CHMP2B GST (Abnova), CHMP7 GST (Abnova), CHMP2B His (MyBioSource), GST (Thermo Fisher Scientific), and His (MyBioSource) were added to 50 µL binding buffer (50 mM Tris pH 7.4, 150 mM NaCl, 2 mM MgCl_2_, 2 mM CaCl_2_, 10% glycerol, 0.5% NP-40, 1 mM DTT, 1X EDTA free protease inhibitor cocktail). For reactions where multiple recombinant proteins were added, proteins were added in a 1:1 ratio (2 µg each) and total reaction volume remained the same. Binding reactions were incubated at 4 °C with end-over-end rotation for 2 h. About 1.5 h into the reaction incubation, magnetic GST or His beads (Thermo Fisher Scientific) were washed 3 × 10 min with binding buffer. Binding buffer wash was removed and appropriate binding reactions were added to washed beads. Reactions were then incubated at 4 °C with end-over-end rotation for an additional 1 h. Following incubation, 25 µL supernatant was collected as the unbound fraction and added to 25 µL 2X Laemmli buffer (BioRad). Remaining supernatant was discarded and protein – bead complexes were washed with binding buffer 3 × 5 min at 4 °C with end-over-end rotation. Washes were discarded and beads were resuspending in 50 µL 1X Laemmli buffer. 20 uL of each sample was subjected to SDS-PAGE using 4–20% acrylamide gels (BioRab). Gels were run at a constant 150 V for approximately 45 min until the dye front reached the end of the gel. Following electrophoresis, gels were washed 3 × 5 min with ddH_2_O and then incubated with SimplyBlue SafeStain (Thermo Fisher Scientific) for 1 h at room temperature with gentle rocking. SimplyBlue stained gels were washed 3 × 1 h with ddH_2_O and protein bands were imaged with the ImageQuant LAS 4000 system (GE Healthcare).

### Statistical analyses

Experimenters were blinded to iPSC line genotype, treatment condition, and time point throughout the duration of the experiment. All data analysis was carried out using FIJI version 2.3.0/1.53q and was completely automated based on automated spot counting and mask based region of interest definition using DAPI and/or Map2 signals. In cases where regions of interest were manually defined, the experimenter remained blinded to genotype, treatment, and time point information to eliminate bias when defining regions of interest in the DAPI and/or Map2 channels. No data points were excluded from analysis in this study. All statistical analyses were performed using GraphPad Prism version 9 and 10 (GraphPad). Statistical analyses were performed as previously described [5, 22, 23] where the average of individual cells or nuclei per iPSC line and treatment condition was calculated to represent *n* = 1. Total n’s (iPSC lines and cells per iPSC lines) are reported in figure legends. Student’s t-test, One-way ANOVA with Tukey’s multiple comparison test, Two-way ANOVA with Tukey’s multiple comparison test, Student’s t-test, or Fisher’s exact test was used as described in figure legends. * *p* < 0.05, ** *p* < 0.01, *** *p* < 0.001, **** *p* < 0.0001. Superplots [54] were used to detail the full spread of variability of large data sets and indicate iPSC line averages (displayed individual data points) used for statistical analyses. Within each violin plot, the center dotted line indicates the median value of all data points within the larger individual cell based data set and the two additional dotted lines indicate the 25th and 75th percentiles. Bar graphs were used to display individual data points with error bars representing +/- SEM from bulk data where individual cell or nuclei quantifications were not performed (e.g. western blot, qRT-PCR).

## Electronic supplementary material

Below is the link to the electronic supplementary material.

Supplementary Material 1

Supplementary Material 2

## Data Availability

No datasets were generated or analysed during the current study.

## Abbreviations

ALS: Amyotrophic Lateral Sclerosis
FTD: Frontotemporal Dementia
fALS: Familial Amyotrophic Lateral Sclerosis
sALS: Sporadic Amyotrophic Lateral Sclerosis
iPSC: Induced Pluripotent Stem Cell
iPSN: Induced Pluripotent Stem Cell Derived Neuron
NPC: Nuclear Pore Complex
Nup: Nucleoporin
NCT: Nucleocytoplasmic Transport
ASO: Antisense Oligonucleotide
RITE: Recombination Induced Tag Exchange
4-OHT: 4-hydroxytamoxifen
NES: Nuclear Export Sequence
MIM: Microtubule Interacting Motif
NT: Non-Targeting
