# Supplementary material for: CHMP2B promotes CHMP7 mediated nuclear pore complex injury in sporadic ALS
Source: Acta Neuropathol Commun. 2024 Dec 21;12:199. doi: 10.1186/s40478-024-01916-7 (PMC11662732; doi:10.1186/s40478-024-01916-7)
Supplement: Supplementary file 1 — Supplementary Material 1 [file 40478_2024_1916_MOESM1_ESM.pdf]

Supplemental Table 1: Demographic Information for iPSC Lines

iPSC Line Name	Source	Clinical Diagnosis	Age at Time of Collection	Sex	Origin
CS0002	Cedars-Sinai	Non-neurologic control	51	Male	PBMC
CS0201	Cedars-Sinai	Non-neurologic control	56	Female	PBMC
CS0202	Cedars-Sinai	Non-neurologic control	57	Male	PBMC
CS0206	Cedars-Sinai	Non-neurologic control	72	Female	PBMC
CS1ATZ	Cedars-Sinai	Non-neurologic control	60	Male	PBMC
CS2AE8	Cedars-Sinai	Non-neurologic control	50	Female	PBMC
CS8PAA	Cedars-Sinai	Non-neurologic control	58	Female	PBMC
CS9XH7	Cedars-Sinai	Non-neurologic control	53	Male	PBMC
EDi022-A	Cedars-Sinai	Non-neurologic control	79	Male	PBMC
EDi034-A	Cedars-Sinai	Non-neurologic control	79	Female	PBMC
EDi036-A	Cedars-Sinai	Non-neurologic control	79	Female	PBMC
EDi037-A	Cedars-Sinai	Non-neurologic control	79	Male	PBMC
EDi043-A	Cedars-Sinai	Non-neurologic control	80	Male	PBMC
EDi044-A	Cedars-Sinai	Non-neurologic control	80	Female	PBMC
CS0JGZ	Cedars-Sinai	sALS	56	Male	PBMC
CS1KL3	Cedars-Sinai	sALS	71	Female	PBMC
CS2EVP	Cedars-Sinai	sALS	69	Male	PBMC
CS3EPR	Cedars-Sinai	sALS	60	Female	PBMC
CS3XLK	Cedars-Sinai	sALS	55	Female	PBMC
CS4KGP	Cedars-Sinai	sALS	82	Female	PBMC
CS4PFR	Cedars-Sinai	sALS	72	Female	PBMC
CS4ZCD	Cedars-Sinai	sALS	53	Male	PBMC
CS5JPF	Cedars-Sinai	sALS	55	Female	PBMC
CS5ZHY	Cedars-Sinai	sALS	64	Male	PBMC
CS6EVH	Cedars-Sinai	sALS	56	Male	PBMC
CS6PYD	Cedars-Sinai	sALS	69	Female	PBMC
CS8JGP	Cedars-Sinai	sALS	61	Male	PBMC
CS9GXD	Cedars-Sinai	sALS	68	Male	PBMC

Supplemental Table 2: ASO Sequences

ASO Name	Sequence
Scrambled (Ionis ID: 676630)	CCTATAGGACTATCCAGGAA
CHMP7 (Ionis ID: 1508917)	TGTTACCCTCAGATACCGCC
CHMP2B ASO 1	TGTGACAAGTCCTAGTGGGA
CHMP2B ASO 2	AGGGTGAGAAAGAGTATGGG
CHMP2B ASO 3	GGGATGCAGAAATACAGAGG
CHMP2B ASO 4	AAGTGGCATCTGAAGTAGGG
CHMP2B ASO 5	TGGTAAGCGAAGATGCCAGA
CHMP2B ASO 6	ATCACTTACCACCTGCCATG
CHMP2B ASO 7	GTTGGTGGGACAGAGCAATA
CHMP2B ASO 8	CACCTAACTCTCTAGTGGCT
CHMP2B ASO 9	TCAATGGAACAGAGCCCAGA
CHMP2B ASO 10	CATTTACCCTATGACCCAGC

Supplemental Table 3: Plasmid Information

Plasmid	Backbone	Source
CHMP7 WT Flag	pcDNA3.1	Vietri et al., 2020
CHMP7 Open Flag	pcDNA3.1	Genscript, This Paper
CHMP7 ∆Helix 6 Flag	pcDNA3.1	Genscript, This Paper
CHMP7 NES1*/NES2* Flag	pcDNA3.1	Genscript, This Paper
POM121 RITE (Myc-Flag)	pLenti CMV/TO Hygro DEST	Toyoma et al., 2018

Supplemental Table 4: Antibody Information

Primary Antibodies			
**Antibody**	**Source**	**Catalog Number**	**Application and Concentration**
Rabbit Anti-CHMP7	ProteinTech	16424-1-AP	IF: 1/250
Mouse Anti-CHMP7	ProteinTech	68406-1-Ig	IF: 1/250
Rabbit Anti-POM121	ProteinTech	15645-1-AP	IF: 1/250
Rabbit Anti-CHMP2B	Cell Signaling	76173S	Western: 1/1000<br>IF: 1/250
Mouse Anti-CHMP2B	Invitrogen	MA5-21591	IF: 1/250
Rabbit Anti-CHMP2B	ProteinTech	12527-1-AP	Western: 1/1000<br>IF: 1/250
Mouse Anti-CHMP2B	R&D Systems	MAB7509	Western: 1/1000
Guinea Pig Anti-Map2	Synaptic Systems	188004	IF: 1/1000
Rat Anti-Flag	Thermo Fisher Scientific	MA1-142	IF: 1/2500
Mouse Anti-Myc	Cell Signaling	2276S	IF: 1/500
Mouse Anti-GAPDH	Life Technologies	AM4300	Western: 1/10,000
**Secondary Antibodies**			
Goat Anti-Mouse Alexa 488	Invitrogen	A11029	IF: 1/1000
Goat Anti-Rabbit Alexa 488	Invitrogen	A11034	IF: 1/1000
Goat Anti-Rat Alexa 488	Invitrogen	A11006	IF: 1/1000
Goat Anti-Mouse Alexa 568	Invitrogen	A11031	IF: 1/1000
Goat Anti-Rabbit Alexa 568	Invitrogen	A11036	IF: 1/1000
Goat Anti-Rat Alexa 568	Invitrogen	A11077	IF: 1/1000
Goat Anti-Guinea Pig Alexa 568	Invitrogen	A11075	IF: 1/1000
Goat Anti-Mouse Alexa 647	Invitrogen	A21236	IF: 1/1000
Goat Anti-Rabbit Alexa 647	Invitrogen	A21245	IF: 1/1000
Goat Anti-Rabbit IgG HRP	Cell Signaling	7074S	Western: 1/5000
Horse Anti-Mouse IgG HRP	Cell Signaling	7076S	Western: 1/5000

Supplemental Table 5: Demographic Information for Postmortem Human Tissue

	**Clinical Diagnosis**	**Age of Death**	**Sex**
**Control-1**	Non-neurologic control	71	Female
**Control-2**	Non-neurologic control	52	Male
**Control-3**	Non-neurologic control	74	Female
**Control-4**	Non-neurologic control	92	Female
**Control-5**	Non-neurologic control	72	Male
**Control-6**	Non-neurologic control	50	Male
**Control-7**	Non-neurologic control	62	Male
**Control-8**	Non-neurologic control	67	Male
**Control-9**	Non-neurologic control	63	Female
**Control-10**	Non-neurologic control	82	Female
**sALS-1**	sALS	67	Female
**sALS-2**	sALS	59	Male
**sALS-3**	sALS	68	Female
**sALS-4**	sALS	69	Male
**sALS-5**	sALS	65	Male
**sALS-6**	sALS	78	Male
**sALS-7**	sALS	68	Female
**sALS-8**	sALS	57	Female
**sALS-9**	sALS	61	Female
**sALS-10**	sALS	51	Male
**sALS-11**	sALS	80	Male
**sALS-12**	sALS	72	Female
**sALS-13**	sALS	70	Female
**sALS-14**	sALS	68	Male
**sALS-15**	sALS	69	Male

Supplemental Table 6: Primer Sequences and TaqMan Probe IDs for qRT-PCR

Primer Sequences		
**Target**	**Sequence**	
STMN2	Forward	AGCTGTCCATGCTGTCACTG
	Reverse	GGTGGCTTCAAGATCAGCTC
Truncated STMN2	Forward	GGACTCGGCAGAAGACCTTC
	Reverse	GCAGGCTGTCTGTCTCTCTC
GAPDH	Forward	GAAGGTGAAGGTCGGAGTC
	Reverse	GAAGATGGTGATGGGATTTC
ACTL6B CE	Forward	ATCCTGGATCACACCTACAGC
	Reverse	AGGAGGATTGCTTGAACCC
ARHGAP32 CE	Forward	CACCTTCTAAATTCTGGTTTTGAAG
	Reverse	CAGATACAGACGAAAAAGCTGAGTT
CAMK2B CE	Forward	GAGTGCAGAGACTTCCCCC
	Reverse	CTGCTCCGTGGTCTTAATGAT
CDK7 CE	Forward	GCAGTGTGGACATGACTGATA
	Reverse	GTCCACACCTACACCATACATC
DNM1 CE	Forward	TGACCCTTTCGGCCCT
	Reverse	CACGAAATCAACATGGCAGT
HDGFL2 CE	Forward	TCACACCTGAGAAGAAAGCAG
	Reverse	TCCTCTCTTCTGTGTCCCTCT
MYO18A CE	Forward	AAGTCCAGGGATGAGATTGTG
	Reverse	GCAGAGTTTTGTCCTCCTCTTTA
SYT7 CE	Forward	GCAGTGAGAAGAAGGCTATCAA
	Reverse	CGGCAGACTGGAGCCT

TaqMan Probe IDs	
**Target**	**TaqMan ID**
ELAVL3	Hs00154959_m1
PFKP	Hs00737347_m1
RCAN1	Hs01120954_m1
SELPLG	Hs05033974_s1
POM121	Hs00208021_m1

Actin	Hs03023943_g1
GAPDH	Hs02786624_g1
