# Supplementary material for: CHMP2B promotes CHMP7 mediated nuclear pore complex injury in sporadic ALS
Source: Acta Neuropathol Commun. 2024 Dec 21;12:199. doi: 10.1186/s40478-024-01916-7 (PMC11662732; doi:10.1186/s40478-024-01916-7)
Supplement: Supplementary file 2 — Supplementary Material 2 [file 40478_2024_1916_MOESM2_ESM.pdf]

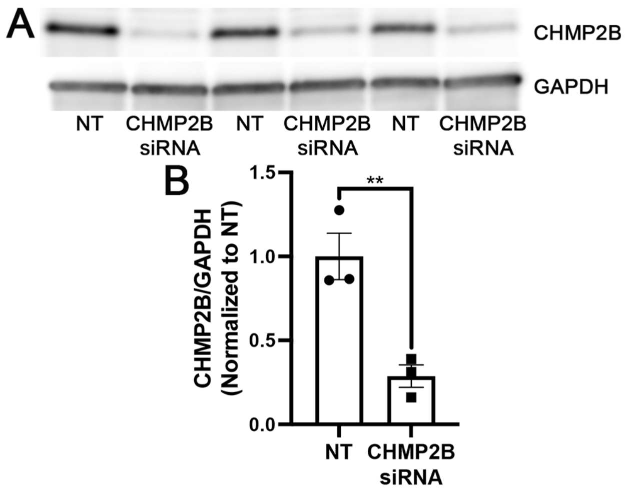

Supplemental Figure 1: siRNA mediated knockdown of CHMP2B in iPSNs. (**A**) Western blot for CHMP2B expression 3 weeks following nucleofection of CHMP2B or non-targeting (NT) control siRNAs. siRNA as indicated on bottom, antibody for western blot as indicated on right. (**B**) Quantification of CHMP2B protein expression in iPSN lysates 3 weeks following the nucleofection of CHMP2B or NT control siRNAs. GAPDH was used for normalization. n = 3 control iPSC lines. Student's t-test was used to calculate statistical significance. ** $p < 0.01$.

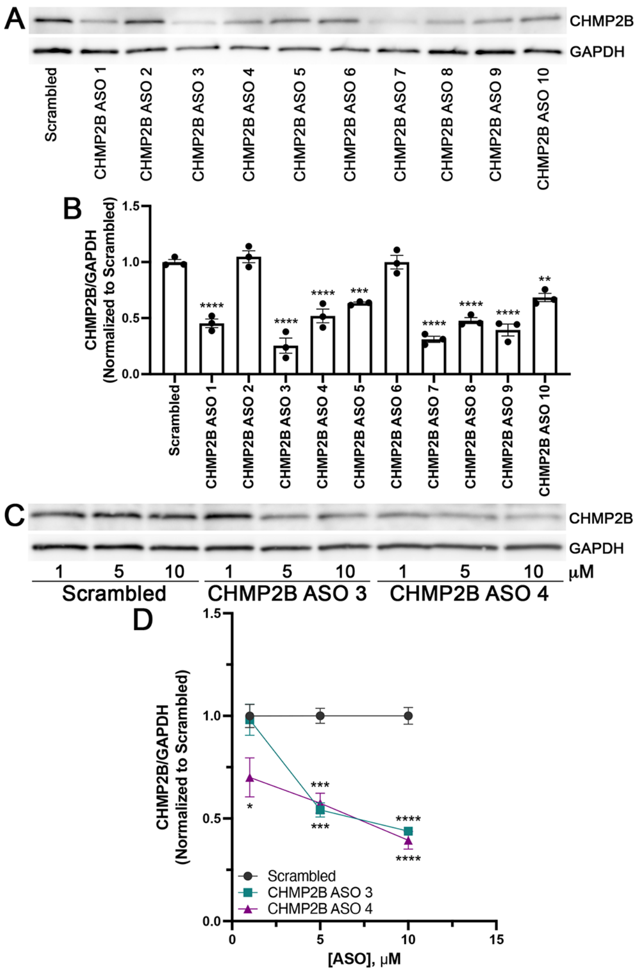

Supplemental Figure 2: ASO mediated knockdown of CHMP2B in iPSNs. (**A**) Western blot for CHMP2B expression following 3 weeks of treatment with 5 μM scrambled control or CHMP2B targeting ASOs. ASO as indicated on bottom, antibody for western blot as indicated on right. (**B**) Quantification of CHMP2B protein expression in iPSN lysates following 3 weeks of treatment with scrambled control or CHMP2B targeting ASOs. GAPDH was used for normalization. n = 3 control iPSC lines. One-way ANOVA with Tukey's multiple comparison test was used to calculate statistical significance. ** $p < 0.01$, *** $p < 0.001$, **** $p < 0.0001$. (**C**) Western blot for CHMP2B expression following 3 weeks of treatment with 1, 5, and 10 μM scrambled control or CHMP2B targeting ASOs. ASO and concentration as indicated on bottom, antibody for western blot as indicated on right. (**D**) Quantification of CHMP2B protein expression in iPSN lysates following 3 weeks of treatment with scrambled control or CHMP2B targeting

ASOs. GAPDH was used for normalization. n = 3 control iPSC lines. Two-way ANOVA with Tukey's multiple comparison test was used to calculate statistical significance. CHMP2B ASO 3 significance compared to scrambled control as indicated on top, CHMP2B ASO 4 significance compared to scrambled control as indicated on bottom ** $p < 0.01$, *** $p < 0.001$, **** $p < 0.0001$.

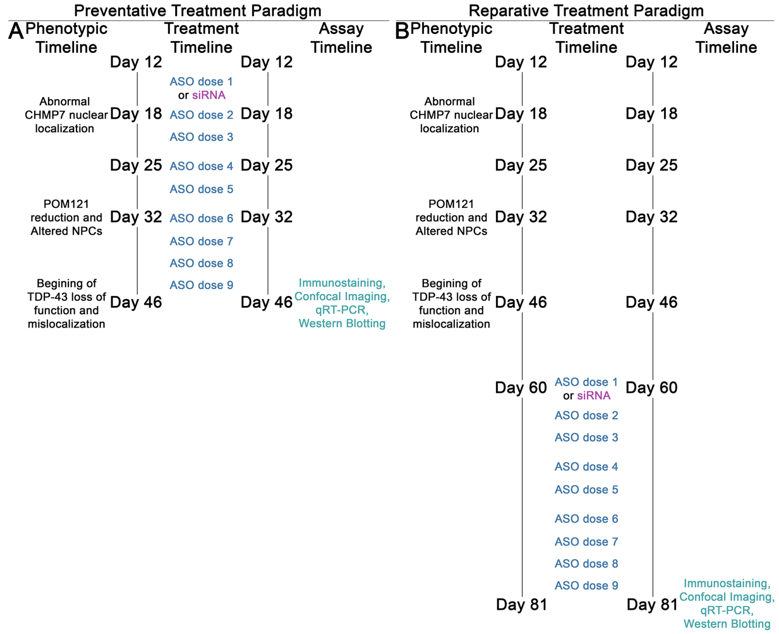

Supplemental Figure 3: Schematic of preventative and reparative siRNA and ASO treatment paradigms. (**A**) Schematic of pathologic events in NPC injury cascades, preventative treatment paradigm, and experimental time points used in this study. (**B**) Schematic of pathologic events in NPC injury cascades, reparative treatment paradigm, and experimental time points used in this study.

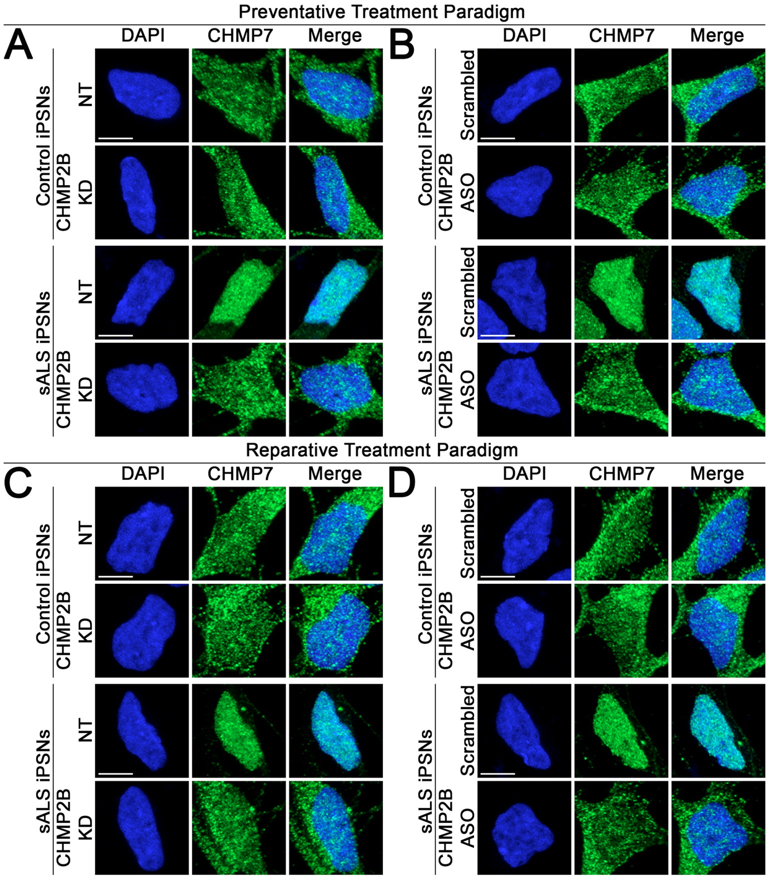

Supplemental Figure 4: High magnification images of CHMP7 in control and sALS iPSNs following knockdown of CHMP2B. (**A**) Maximum intensity projections from immunostaining and confocal imaging of CHMP7 in control and sALS iPSNs on day 46 of differentiation (3 weeks following nucleofection of CHMP2B or non-targeting (NT) siRNAs). siRNA and genotype as indicated on left, antibodies for immunostaining as indicated on top. Knockdown was initiated at day 15 of differentiation (the time point where nuclear localization of CHMP7 begins to increase in sALS iPSNs [20]). (**B**) Maximum intensity projections from immunostaining and confocal imaging of CHMP7 in control and sALS iPSNs on day 46 of differentiation following 3 weeks of treatment with scrambled control of CHMP2B targeting ASOs. ASO and genotype as indicated on left, antibodies for immunostaining as indicated on top. ASO treatment was initiated at day 15 of differentiation (the time point where nuclear localization of CHMP7 begins to increase in sALS iPSNs [20]). (**C**) Maximum intensity projections from immunostaining and

confocal imaging of CHMP7 in control and sALS iPSNs on day 81 of differentiation (3 weeks following nucleofection of CHMP2B or non-targeting (NT) siRNAs). siRNA and genotype as indicated on left, antibodies for immunostaining as indicated on top. Knockdown was initiated at day 60 of differentiation following the emergence of NPC injury and TDP-43 loss of function and mislocalization [20, 82]. (**D**) Maximum intensity projections from immunostaining and confocal imaging of CHMP7 in control and sALS iPSNs on day 81 of differentiation following 3 weeks of treatment with scrambled control of CHMP2B targeting ASOs. ASO and genotype as indicated on left, antibodies for immunostaining as indicated on top. ASO treatment was initiated at day 60 of differentiation following the emergence of NPC injury and TDP-43 loss of function and mislocalization [20, 82]. Scale bar = 5 μm.

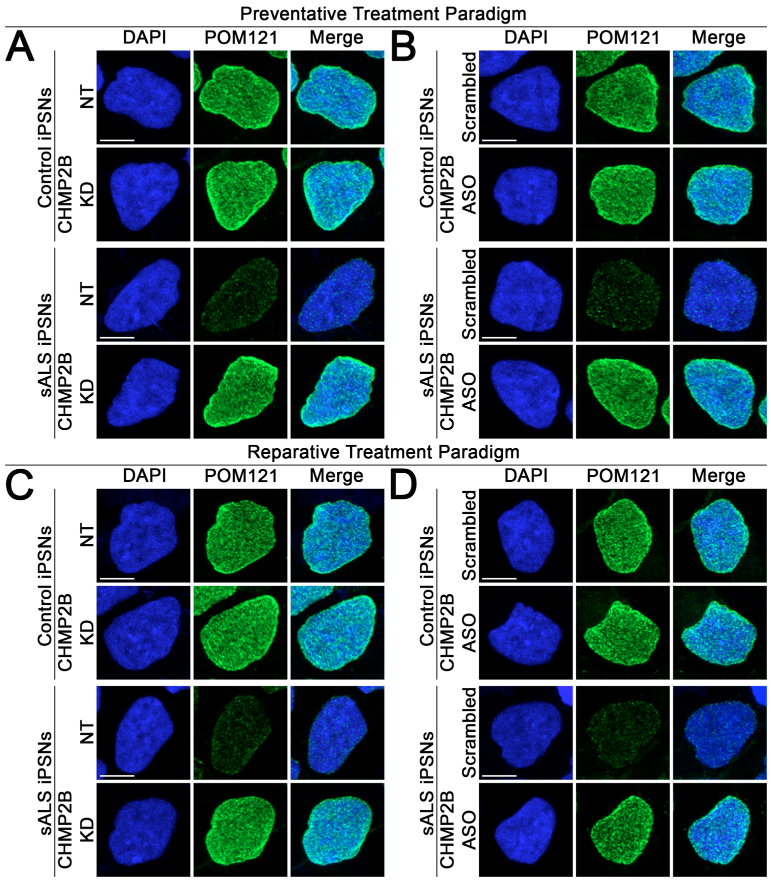

Supplemental Figure 5: High magnification images of POM121 in control and sALS iPSNs following knockdown of CHMP2B. (**A**) Maximum intensity projections from immunostaining and confocal imaging of POM121 in control and sALS iPSNs on day 46 of differentiation (3 weeks following nucleofection of CHMP2B or non-targeting (NT) siRNAs). siRNA and genotype as indicated on left, antibodies for immunostaining as indicated on top. Knockdown was initiated at day 15 of differentiation (the time point where nuclear localization of CHMP7 begins to increase in sALS iPSNs [20]). (**B**) Maximum intensity projections from immunostaining and confocal imaging of POM121 in control and sALS iPSNs on day 46 of differentiation following 3 weeks of treatment with scrambled control of CHMP2B targeting ASOs. ASO and genotype as indicated on left, antibodies for immunostaining as indicated on top. ASO treatment was initiated at day 15 of differentiation (the time point where nuclear localization of CHMP7 begins to increase in sALS iPSNs [20]). (**C**) Maximum intensity projections from immunostaining and

confocal imaging of POM121 in control and sALS iPSNs on day 81 of differentiation (3 weeks following nucleofection of CHMP2B or non-targeting (NT) siRNAs). siRNA and genotype as indicated on left, antibodies for immunostaining as indicated on top. Knockdown was initiated at day 60 of differentiation following the emergence of NPC injury and TDP-43 loss of function and mislocalization [20, 82]. (**D**) Maximum intensity projections from immunostaining and confocal imaging of POM121 in control and sALS iPSNs on day 81 of differentiation following 3 weeks of treatment with scrambled control of CHMP2B targeting ASOs. ASO and genotype as indicated on left, antibodies for immunostaining as indicated on top. ASO treatment was initiated at day 61 of differentiation following the emergence of NPC injury and TDP-43 loss of function and mislocalization [20, 82]. Scale bar = 5 μm.

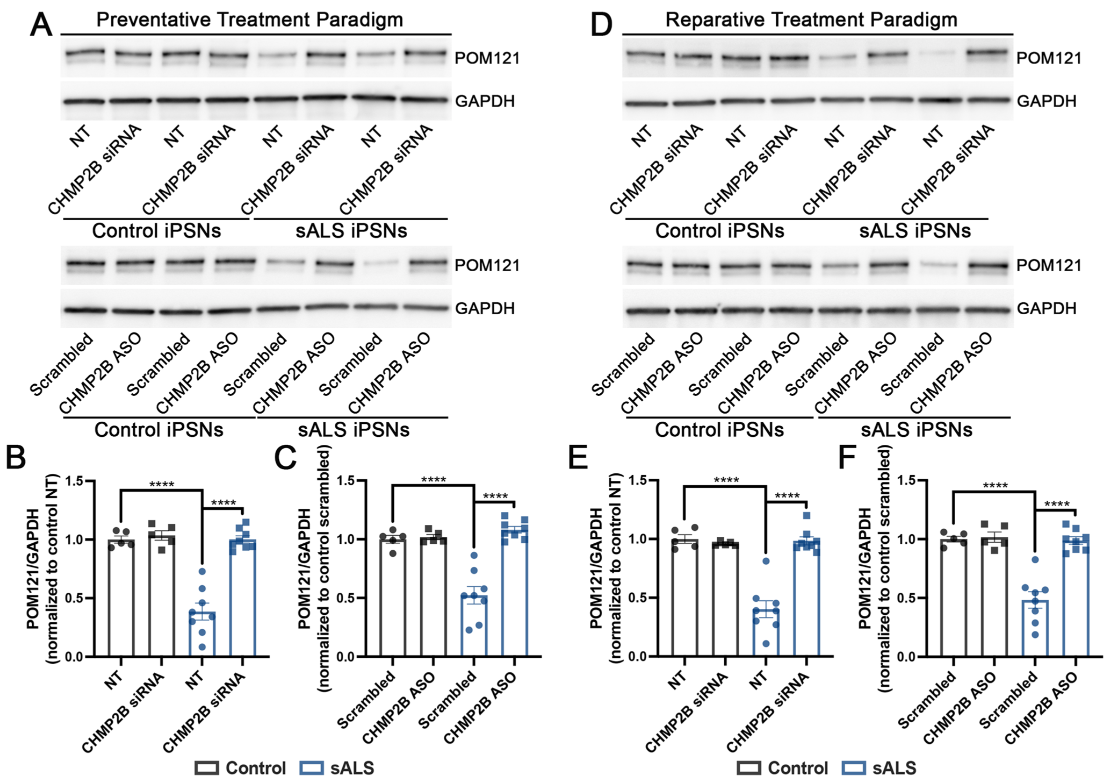

Supplemental Figure 6: siRNA and ASO mediated knockdown of CHMP2B restores levels of POM121 in sALS iPSN nuclei. (**A**) Western blot for POM121 expression in nuclei isolated on day 46 of differentiation 3 weeks following nucleofection of CHMP2B or non-targeting (NT) control siRNAs or following 3 weeks of treatment with 5 μM scrambled control or CHMP2B targeting ASOs. Genotype as indicated on top, siRNA and ASO as indicated on bottom, antibody for western blot as indicated on right. siRNA nucleofection or ASO treatment was initiated at day 15 of differentiation (the time point where nuclear localization of CHMP7 begins to increase in sALS iPSNs [20]). (**B-C**) Quantification of POM121 protein expression in iPSN nuclei lysates 3 weeks following the nucleofection of CHMP2B or NT control siRNAs (**B**) or following 3 weeks treatment with scrambled control or CHMP2B targeting ASOs (**C**). GAPDH was used for normalization. n = 5 control and 8 sALS iPSC lines. Tukey's multiple comparison test was used to calculate statistical significance. **** $p < 0.0001$. (**D**) Western blot for POM121 expression in nuclei isolated on day 81 of differentiation 3 weeks following nucleofection of CHMP2B or non-targeting (NT) control siRNAs or following 3 weeks of treatment with 5 μM

scrambled control or CHMP2B targeting ASOs. Genotype as indicated on top, siRNA and ASO as indicated on bottom, antibody for western blot as indicated on right. siRNA nucleofection or ASO treatment was initiated at day 60 of differentiation following the emergence of NPC injury and TDP-43 loss of function and mislocalization [20, 82]. (**E-F**) Quantification of POM121 protein expression in iPSN nuclei lysates 3 weeks following the nucleofection of CHMP2B or NT control siRNAs (**E**) or following 3 weeks treatment with scrambled control or CHMP2B targeting ASOs (**F**). GAPDH was used for normalization. n = 5 control and 8 sALS iPSC lines. Tukey's multiple comparison test was used to calculate statistical significance. **** $p < 0.0001$.

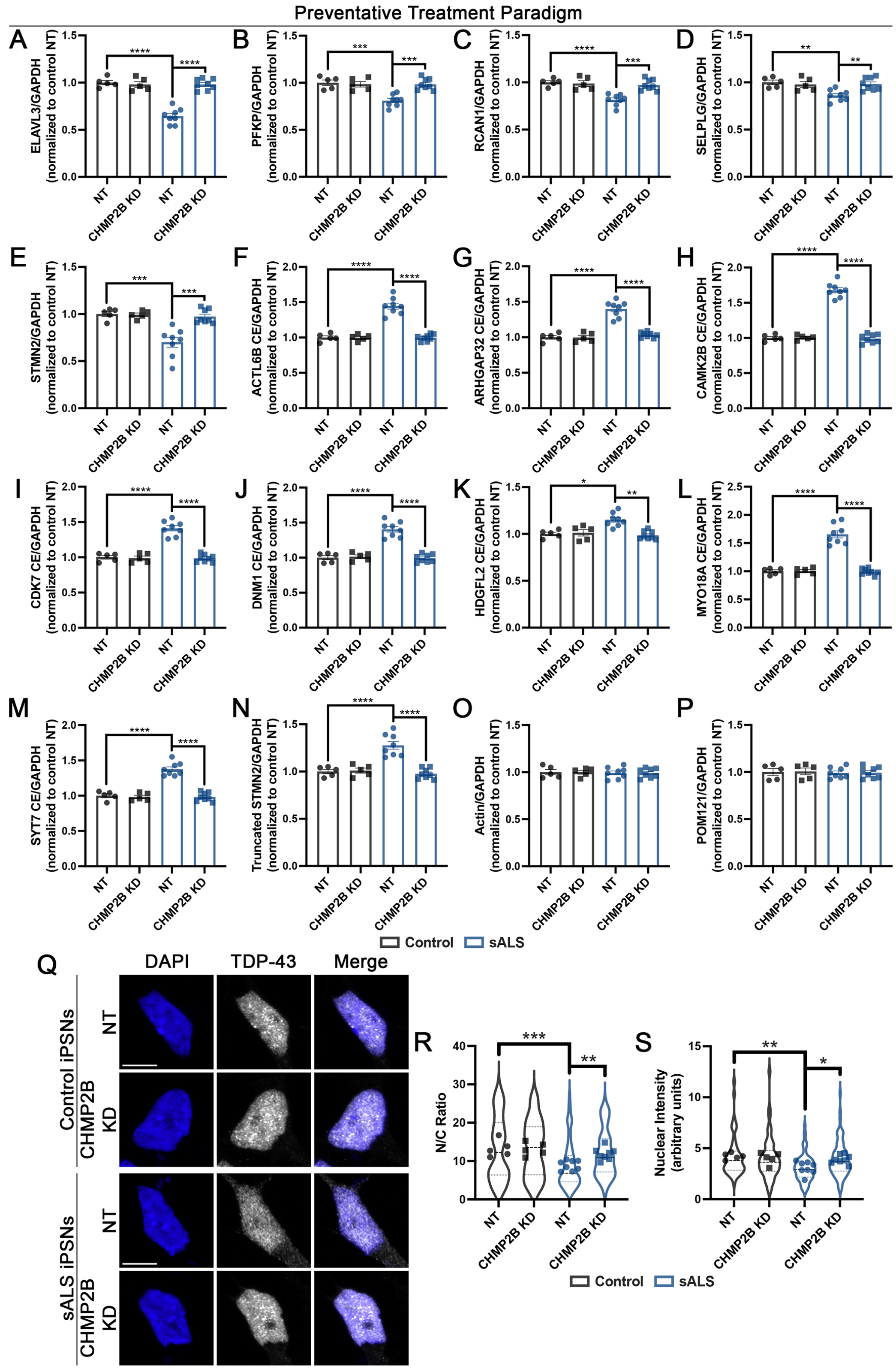

Supplemental Figure 7: Early siRNA mediated knockdown of CHMP2B restores TDP-43 function in sALS iPSNs. (**A-P**) qRT-PCR for *ELAVL3* (**A**), *PFKP* (**B**), *RCAN1* (**C**), *SELPLG* (**D**),

and *STMN2* (**E**), *ACTL6B* (**F**), *ARHGAP32* (**G**), *CAMK2B* (**H**), *CDK7* (**I**), *DNM1* (**J**), *HDGFL2* (**K**), *MYO18A* (**L**), and *SYT7* (**M**) cryptic exon (CE) containing mRNA, and Truncated *STMN2* (**N**), *Actin* (**O**), and *POM121* (**P**) mRNA in control and sALS iPSNs on day 46 of differentiation (3 weeks following nucleofection of CHMP2B or non-targeting (NT) siRNAs). Knockdown was initiated at day 15 of differentiation (the time point where nuclear localization of CHMP7 begins to increase in sALS iPSNs [20]). GAPDH was used for normalization. *Actin* and *POM121* were used as negative control mRNAs not known to be regulated by TDP-43. n = 5 control and 8 sALS iPSC lines. Two-way ANOVA with Tukey's multiple comparison test was used to calculate statistical significance. * $p < 0.05$, ** $p < 0.01$, *** $p < 0.001$, **** $p < 0.0001$. (**Q**) Maximum intensity projections and confocal imaging of TDP-43 in control and sALS iPSNs on day 46 of differentiation (3 weeks following nucleofection of CHMP2B or non-targeting (NT) siRNAs). siRNA and genotype as indicated on left, antibodies for immunostaining as indicated on top. Knockdown was initiated at day 15 of differentiation (the time point where nuclear localization of CHMP7 begins to increase in sALS iPSNs [20]). (**R**) Quantification of the nuclear to cytoplasmic ratio of TDP-43. n = 5 control and 8 sALS iPSC lines, 100 Map2+ cells per line/knockdown. Two-way ANOVA with Tukey's multiple comparison test was used to calculate statistical significance. **** $p < 0.0001$. (**S**) Quantification of TDP-43 nuclear intensity. n = 5 control and 8 sALS iPSC lines, 100 Map2+ cells per line/knockdown. Two-way ANOVA with Tukey's multiple comparison test was used to calculate statistical significance. * $p < 0.05$, ** $p < 0.01$, *** $p < 0.001$. Scale bar = 5 μm.

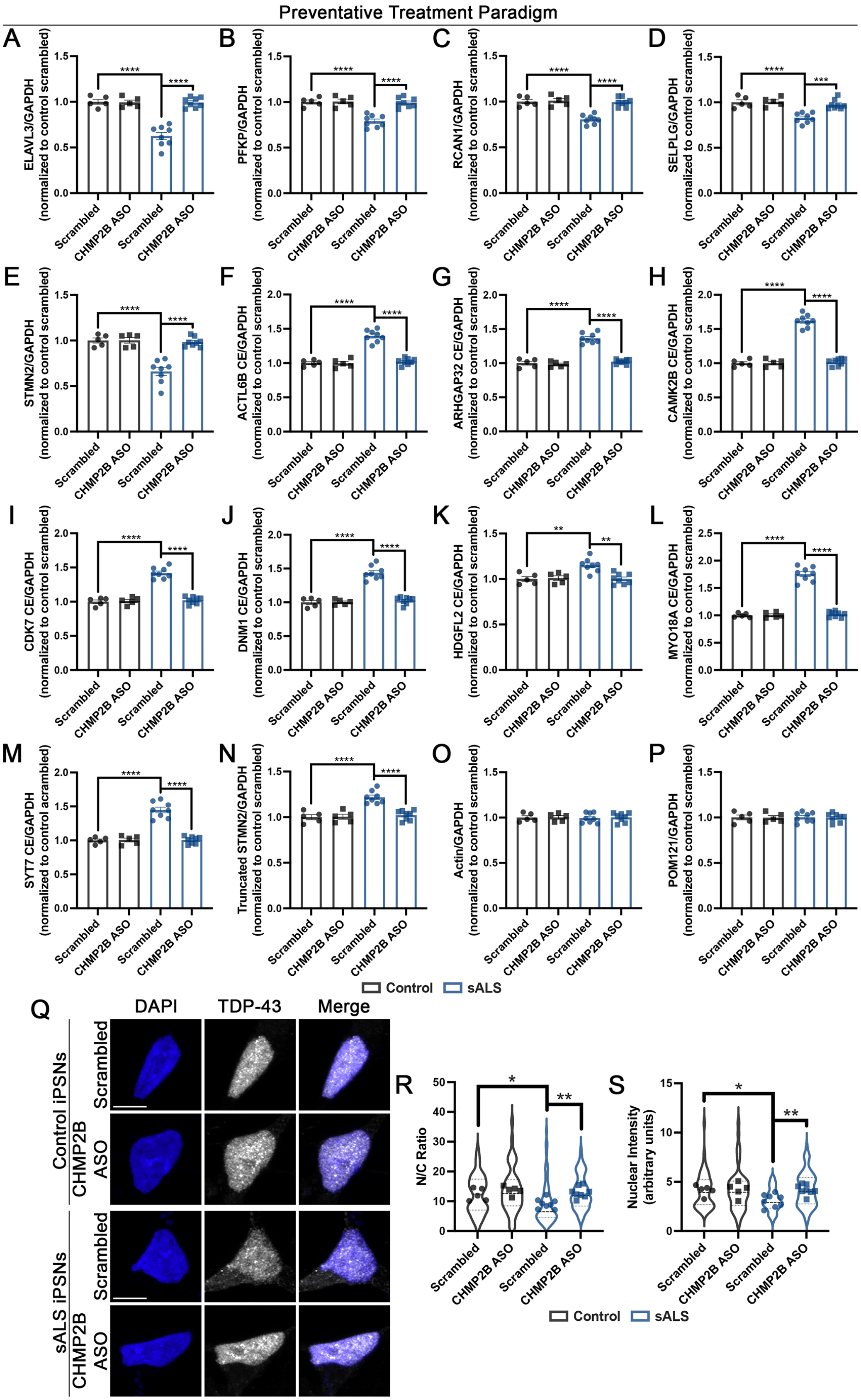

Preventative Treatment Paradigm
A
B
C
D
E
F
G
H
I
J
K
L
M
N
O
P
ELAVL3/GAPDH
PFKP/GAPDH
RCAN1/GAPDH
SELPLG/GAPDH
STMN2/GAPDH
ACTL6B CE/GAPDH
ARHGAP32 CE/GAPDH
CAMK2B CE/GAPDH
CDK7 CE/GAPDH
DNM1 CE/GAPDH
HDGFL2 CE/GAPDH
MYO18A CE/GAPDH
SYT7 CE/GAPDH
Truncated STMN2/GAPDH
Actin/GAPDH
POM121/GAPDH
(normalized to control scrambled)
Scrambled
CHMP2B ASO
Control
sALS
Q
DAPI
TDP-43
Merge
Control iPSNs
sALS iPSNs
R
S
N/C Ratio
Nuclear Intensity (arbitrary units)

Supplemental Figure 8: Early ASO mediated knockdown of CHMP2B restores TDP-43 function in sALS iPSNs. (**A-P**) qRT-PCR for *ELAVL3* (**A**), *PFKP* (**B**), *RCAN1* (**C**), *SELPLG* (**D**), and *STMN2* (**E**), *ACTL6B* (**F**), *ARHGAP32* (**G**), *CAMK2B* (**H**), *CDK7* (**I**), *DNM1* (**J**), *HDGFL2* (**K**), *MYO18A* (**L**), and *SYT7* (**M**) cryptic exon (CE) containing mRNA, and Truncated *STMN2* (**N**), *Actin* (**O**), and *POM121* (**P**) mRNA in control and sALS iPSNs on day 46 of differentiation following 3 weeks of treatment with scrambled control of CHMP2B targeting ASOs. ASO treatment was initiated at day 15 of differentiation (the time point where nuclear localization of CHMP7 begins to increase in sALS iPSNs [20]). GAPDH was used for normalization. *Actin* and *POM121* were used as negative control mRNAs not known to be regulated by TDP-43. n = 5 control and 8 sALS iPSC lines. Two-way ANOVA with Tukey's multiple comparison test was used to calculate statistical significance. ** $p < 0.01$, **** $p < 0.0001$. (**Q**) Maximum intensity projections and confocal imaging of TDP-43 in control and sALS iPSNs on day 46 of differentiation following 3 weeks of treatment with scrambled control of CHMP2B targeting ASOs. ASO and genotype as indicated on left, antibodies for immunostaining as indicated on top. ASO treatment was initiated at day 15 of differentiation (the time point where nuclear localization of CHMP7 begins to increase in sALS iPSNs [20]). (**R**) Quantification of the nuclear to cytoplasmic ratio of TDP-43. n = 5 control and 8 sALS iPSC lines, 100 Map2+ cells per line/knockdown. Two-way ANOVA with Tukey's multiple comparison test was used to calculate statistical significance. **** $p < 0.0001$. (**S**) Quantification of TDP-43 nuclear intensity. n = 5 control and 8 sALS iPSC lines, 100 Map2+ cells per line/knockdown. Two-way ANOVA with Tukey's multiple comparison test was used to calculate statistical significance. * $p < 0.05$, ** $p < 0.01$. Scale bar = 5 μm.

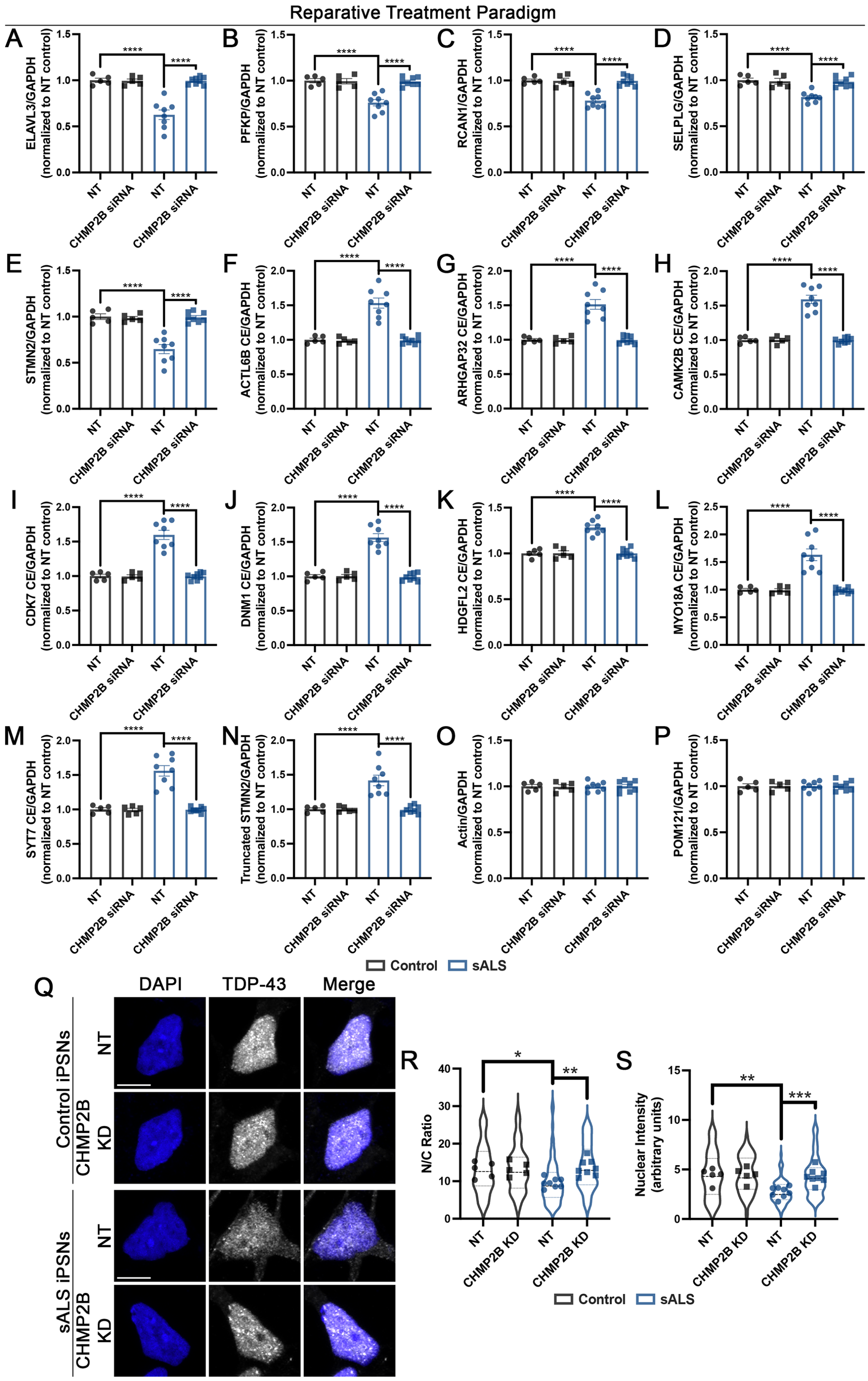

Reparative Treatment Paradigm
A
ELAVL3/GAPDH (normalized to NT control)
B
PFKP/GAPDH (normalized to NT control)
C
RCAN1/GAPDH (normalized to NT control)
D
SELPLG/GAPDH (normalized to NT control)
E
STMN2/GAPDH (normalized to NT control)
F
ACTL6B CE/GAPDH (normalized to NT control)
G
ARHGAP32 CE/GAPDH (normalized to NT control)
H
CAMK2B CE/GAPDH (normalized to NT control)
I
CDK7 CE/GAPDH (normalized to NT control)
J
DNM1 CE/GAPDH (normalized to NT control)
K
HDGFL2 CE/GAPDH (normalized to NT control)
L
MYO18A CE/GAPDH (normalized to NT control)
M
SYT7 CE/GAPDH (normalized to NT control)
N
Truncated STMN2/GAPDH (normalized to NT control)
O
Actin/GAPDH (normalized to NT control)
P
POM121/GAPDH (normalized to NT control)
NT
CHMP2B siRNA
****
Control
sALS
Q
DAPI
TDP-43
Merge
Control iPSNs
sALS iPSNs
NT
CHMP2B KD
R
N/C Ratio
S
Nuclear Intensity (arbitrary units)
*
**
***

Supplemental Figure 9: Late stage siRNA mediated knockdown of CHMP2B restores TDP-43 function in sALS iPSNs. (**A-P**) qRT-PCR for *ELAVL3* (**A**), *PFKP* (**B**), *RCAN1* (**C**), *SELPLG* (**D**), and *STMN2* (**E**), *ACTL6B* (**F**), *ARHGAP32* (**G**), *CAMK2B* (**H**), *CDK7* (**I**), *DNM1* (**J**), *HDGFL2* (**K**), *MYO18A* (**L**), and *SYT7* (**M**) cryptic exon (CE) containing mRNA, and Truncated *STMN2* (**N**), *Actin* (**O**), and *POM121* (**P**) mRNA in control and sALS iPSNs on day 81 of differentiation (3 weeks following nucleofection of CHMP2B or non-targeting (NT) siRNAs). Knockdown was initiated at day 60 of differentiation following the emergence of NPC injury and TDP-43 loss of function and mislocalization [20, 82]. GAPDH was used for normalization. *Actin* and *POM121* were used as negative control mRNAs not known to be regulated by TDP-43. n = 5 control and 8 sALS iPSC lines. Two-way ANOVA with Tukey's multiple comparison test was used to calculate statistical significance. * $p < 0.05$, ** $p < 0.01$, *** $p < 0.001$, **** $p < 0.0001$. (**Q**) Maximum intensity projections and confocal imaging of TDP-43 in control and sALS iPSNs on day 81 of differentiation (3 weeks following nucleofection of CHMP2B or non-targeting (NT) siRNAs). siRNA and genotype as indicated on left, antibodies for immunostaining as indicated on top. Knockdown was initiated at day 60 of differentiation following the emergence of NPC injury and TDP-43 loss of function and mislocalization [20, 82]. (**R**) Quantification of the nuclear to cytoplasmic ratio of TDP-43. n = 5 control and 8 sALS iPSC lines, 100 Map2+ cells per line/knockdown. Two-way ANOVA with Tukey's multiple comparison test was used to calculate statistical significance. **** $p < 0.0001$. (**S**) Quantification of TDP-43 nuclear intensity. n = 5 control and 8 sALS iPSC lines, 100 Map2+ cells per line/knockdown. Two-way ANOVA with Tukey's multiple comparison test was used to calculate statistical significance. * $p <0.05$, ** $p < 0.01$, *** $p < 0.001$. Scale bar = 5 μm.

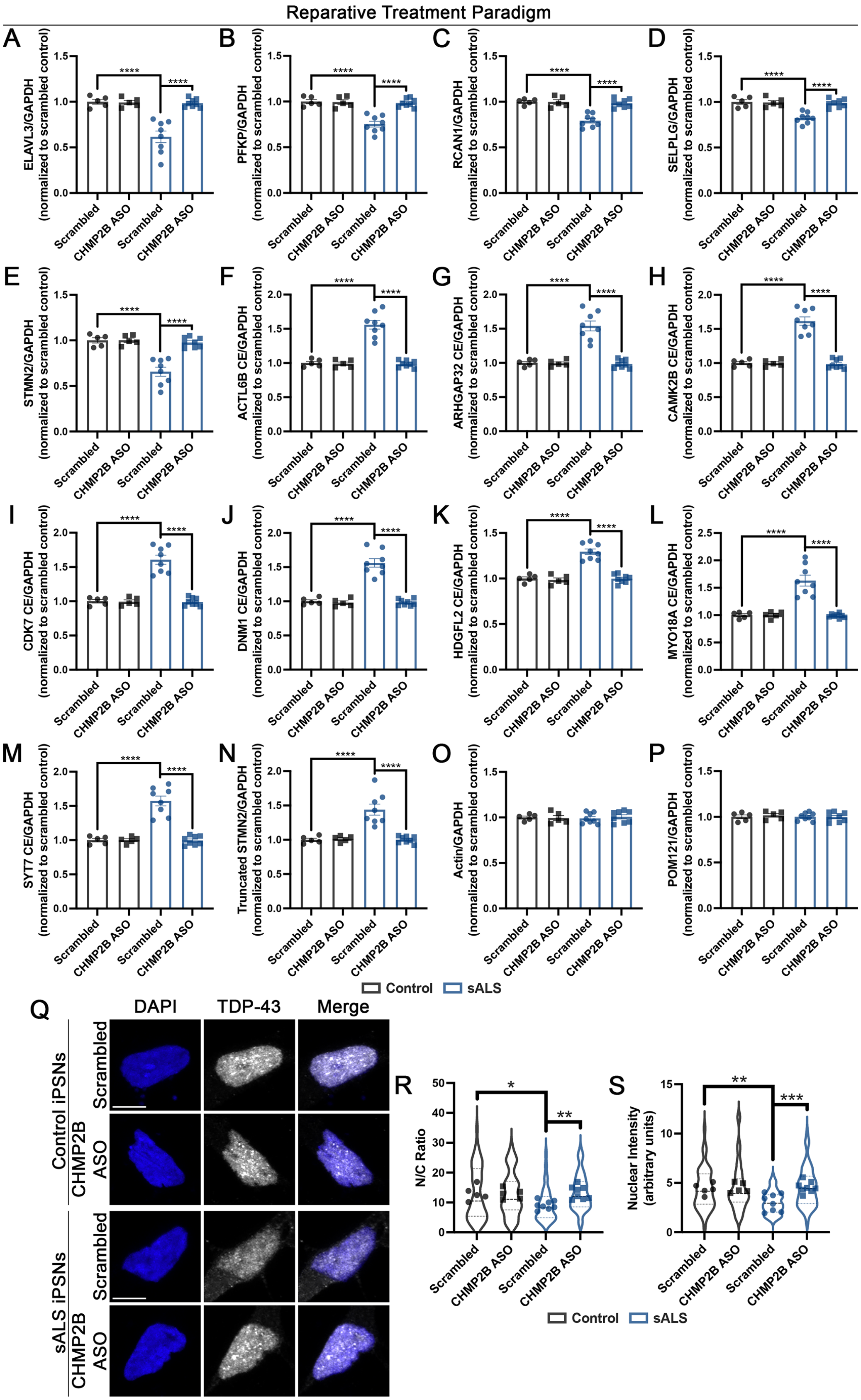

Reparative Treatment Paradigm
A
B
C
D
E
F
G
H
I
J
K
L
M
N
O
P
ELAVL3/GAPDH
PFKP/GAPDH
RCAN1/GAPDH
SELPLG/GAPDH
STMN2/GAPDH
ACTL6B CE/GAPDH
ARHGAP32 CE/GAPDH
CAMK2B CE/GAPDH
CDK7 CE/GAPDH
DNM1 CE/GAPDH
HDGFL2 CE/GAPDH
MYO18A CE/GAPDH
SYT7 CE/GAPDH
Truncated STMN2/GAPDH
Actin/GAPDH
POM121/GAPDH
(normalized to scrambled control)
Scrambled
CHMP2B ASO
Control
sALS
Q
DAPI
TDP-43
Merge
Control iPSNs
sALS iPSNs
R
N/C Ratio
S
Nuclear Intensity
(arbitrary units)

Supplemental Figure 10: Late stage ASO mediated knockdown of CHMP2B restores TDP-43 function in sALS iPSNs. (**A-P**) qRT-PCR for *ELAVL3* (**A**), *PFKP* (**B**), *RCAN1* (**C**), *SELPLG* (**D**), and *STMN2* (**E**), *ACTL6B* (**F**), *ARHGAP32* (**G**), *CAMK2B* (**H**), *CDK7* (**I**), *DNM1* (**J**), *HDGFL2* (**K**), *MYO18A* (**L**), and *SYT7* (**M**) cryptic exon (CE) containing mRNA, and Truncated *STMN2* (**N**), *Actin* (**O**), and *POM121* (**P**) mRNA in control and sALS iPSNs on day 81 of differentiation following 3 weeks of treatment with scrambled control of CHMP2B targeting ASOs. ASO treatment was initiated at day 60 of differentiation following the emergence of NPC injury and TDP-43 loss of function and mislocalization [20, 82]. GAPDH was used for normalization. *Actin* and *POM121* were used as negative control mRNAs not known to be regulated by TDP-43. n = 5 control and 8 sALS iPSC lines. Two-way ANOVA with Tukey's multiple comparison test was used to calculate statistical significance. ** $p < 0.01$, **** $p < 0.0001$. (**Q**) Maximum intensity projections and confocal imaging of TDP-43 in control and sALS iPSNs on day 81 of differentiation following 3 weeks of treatment with scrambled control of CHMP2B targeting ASOs. ASO and genotype as indicated on left, antibodies for immunostaining as indicated on top. ASO treatment was initiated at day 60 of differentiation following the emergence of NPC injury and TDP-43 loss of function and mislocalization [20, 82]. (**R**) Quantification of the nuclear to cytoplasmic ratio of TDP-43. n = 5 control and 8 sALS iPSC lines, 100 Map2+ cells per line/knockdown. Two-way ANOVA with Tukey's multiple comparison test was used to calculate statistical significance. **** $p < 0.0001$. (**S**) Quantification of TDP-43 nuclear intensity. n = 5 control and 8 sALS iPSC lines, 100 Map2+ cells per line/knockdown. Two-way ANOVA with Tukey's multiple comparison test was used to calculate statistical significance. * $p < 0.05$, ** $p < 0.01$, *** $p <0.001$. Scale bar = 5 μm.

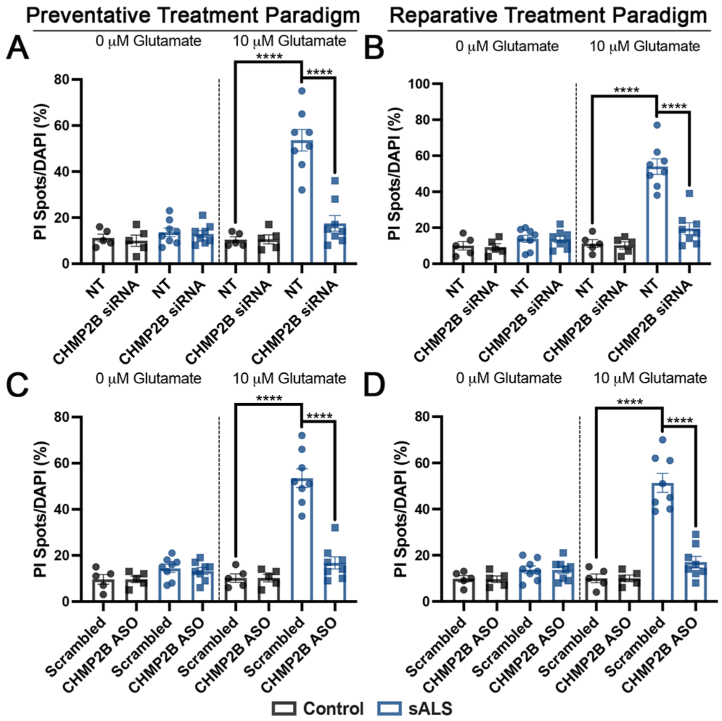

Supplemental Figure 11: siRNA and ASO mediated reduction of CHMP2B alleviates glutamate induced neuronal death in sALS iPSNs. (**A**) Quantification of the percentage of propidium iodide (PI) positive spots normalized to DAPI positive nuclei following 4 hour exposure to 0 or 10 μM glutamate on day 46 of differentiation (3 weeks following nucleofection of CHMP2B or non-targeting (NT) siRNAs). Knockdown was initiated at day 15 of differentiation (the time point where nuclear localization of CHMP7 begins to increase in sALS iPSNs [20]). n = 5 control and 8 sALS iPSC lines. Data points represent the average cell death across 10 images per well for each line/siRNA. Two-way ANOVA with Tukey's multiple comparison test was used to calculate statistical significance. **** $p < 0.0001$. (**B**) Quantification of the percentage of propidium iodide (PI) positive spots normalized to DAPI positive nuclei following 4 hour exposure to 0 or 10 μM glutamate on day 46 of differentiation following 3 weeks of treatment with scrambled control of CHMP2B targeting ASOs. ASO treatment was initiated at day 15 of differentiation (the time point where nuclear localization of CHMP7 begins to increase in sALS iPSNs [20]). n = 5 control and 8 sALS iPSC lines. Data points represent the average cell death

across 10 images per well for each line/ASO. Two-way ANOVA with Tukey's multiple comparison test was used to calculate statistical significance. **** $p < 0.0001$. (**C**) Quantification of the percentage of propidium iodide (PI) positive spots normalized to DAPI positive nuclei following 4 hour exposure to 0 or 10 μM glutamate on day 81 of differentiation (3 weeks following nucleofection of CHMP2B or non-targeting (NT) siRNAs). Knockdown was initiated at day 60 of differentiation following the emergence of NPC injury and TDP-43 loss of function and mislocalization [20, 82]. n = 5 control and 8 sALS iPSC lines. Data points represent the average cell death across 10 images per well for each line/siRNA. Two-way ANOVA with Tukey's multiple comparison test was used to calculate statistical significance. **** $p < 0.0001$. (**D**) Quantification of the percentage of propidium iodide (PI) positive spots normalized to DAPI positive nuclei following 4 hour exposure to 0 or 10 μM glutamate on day 46 of differentiation following 3 weeks of treatment with scrambled control of CHMP2B targeting ASOs. ASO treatment was initiated at day 60 of differentiation following the emergence of NPC injury and TDP-43 loss of function and mislocalization [20, 82]. n = 5 control and 8 sALS iPSC lines. Data points represent the average cell death across 10 images per well for each line/ASO. Two-way ANOVA with Tukey's multiple comparison test was used to calculate statistical significance. **** $p < 0.0001$.

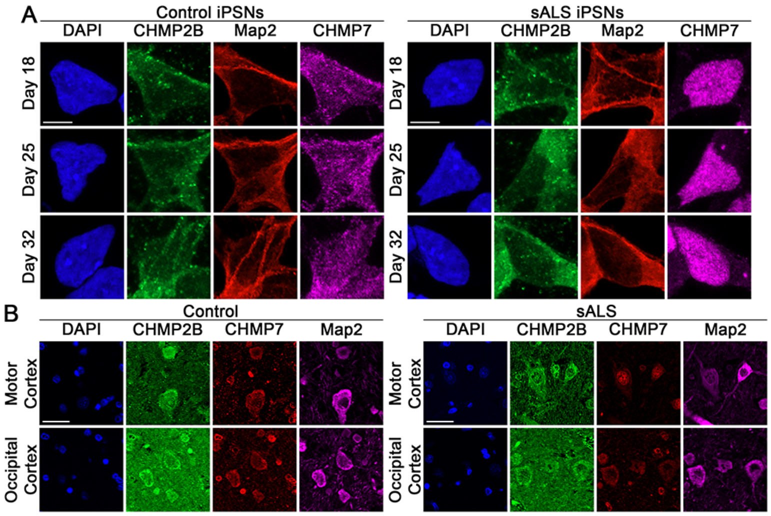

Supplemental Figure 12: Localization of CHMP2B and CHMP7 in control and sALS iPSNs and postmortem human tissues. (**A**) Maximum intensity projections from immunostaining and confocal imaging of CHMP7 and CHMP2B in control and sALS iPSNs. Time points as indicated on left, antibody for immunostaining and genotype as indicated on top. (**B**) Immunostaining and apotome based fluorescent imaging of CHMP7 and CHMP2B in control and sALS postmortem human tissues. Brain region as indicated on left, antibody for immunostaining and genotype as indicated on top.

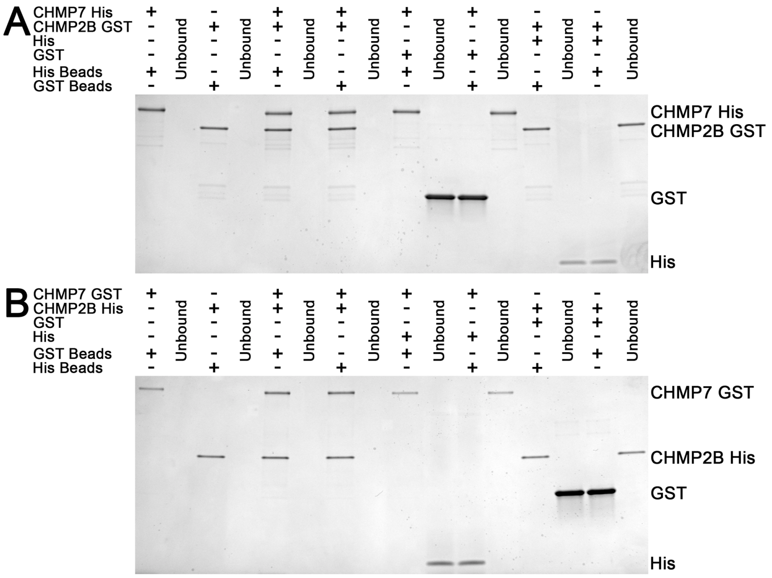

Supplemental Figure 13: CHMP7 and CHMP2B bind *in vitro*. (**A**) Coomassie staining following *in vitro* binding of recombinant His tagged CHMP7 and GST tagged CHMP2B proteins. Binding reactions as indicated on top, recombinant proteins as indicated on right. This experiment was repeated a total of three times. (**B**) Coomassie staining following *in vitro* binding of recombinant GST tagged CHMP7 and His tagged CHMP2B proteins. Binding reactions as indicated on top, recombinant proteins as indicated on right. This experiment was repeated a total of three times.

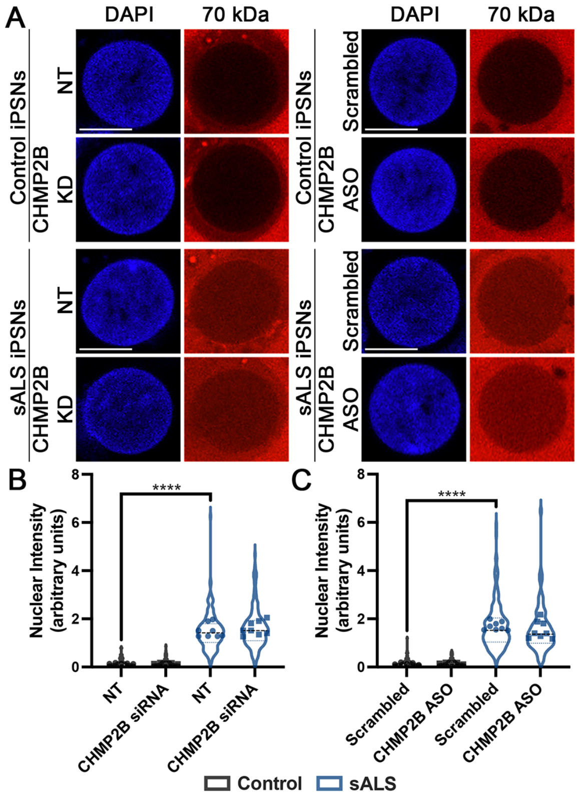

Supplemental Figure 14: Knockdown of CHMP2B does not restore NPC permeability barrier integrity in sALS iPSNs. (**A**) Single z sections from confocal imaging of fluorescently tagged 70 kDa dextrans in digitonin permeabilized control and sALS iPSNs at day 46 of differentiation following 3 weeks of treatment with non-targeting or CHMP2B siRNAs (left) or scrambled control or CHMP2B targeting ASOs (right). Genotype and treatment as indicated on left, dextran as indicated on top. DAPI was used to define the nucleus. (**B**) Quantification of nuclear intensity of 70 kDa dextran in non-targeting and CHMP2B siRNA treated control and sALS iPSNs. n = 5 control and 8 sALS iPSC lines, at least 50 nuclei per line/dextran. Two-way ANOVA with Tukey's multiple comparison test was used to calculate statistical significance. **** $p < 0.0001$. (**C**) Quantification of nuclear intensity of 70 kDa dextran in scrambled control and CHMP2B targeting ASO treated control and sALS iPSNs. n = 5 control and 8 sALS iPSC lines, at least 100 nuclei per line/dextran. Two-way ANOVA with Tukey's multiple comparison test was used to calculate statistical significance. **** $p < 0.0001$. Scale bar = 10 μm.

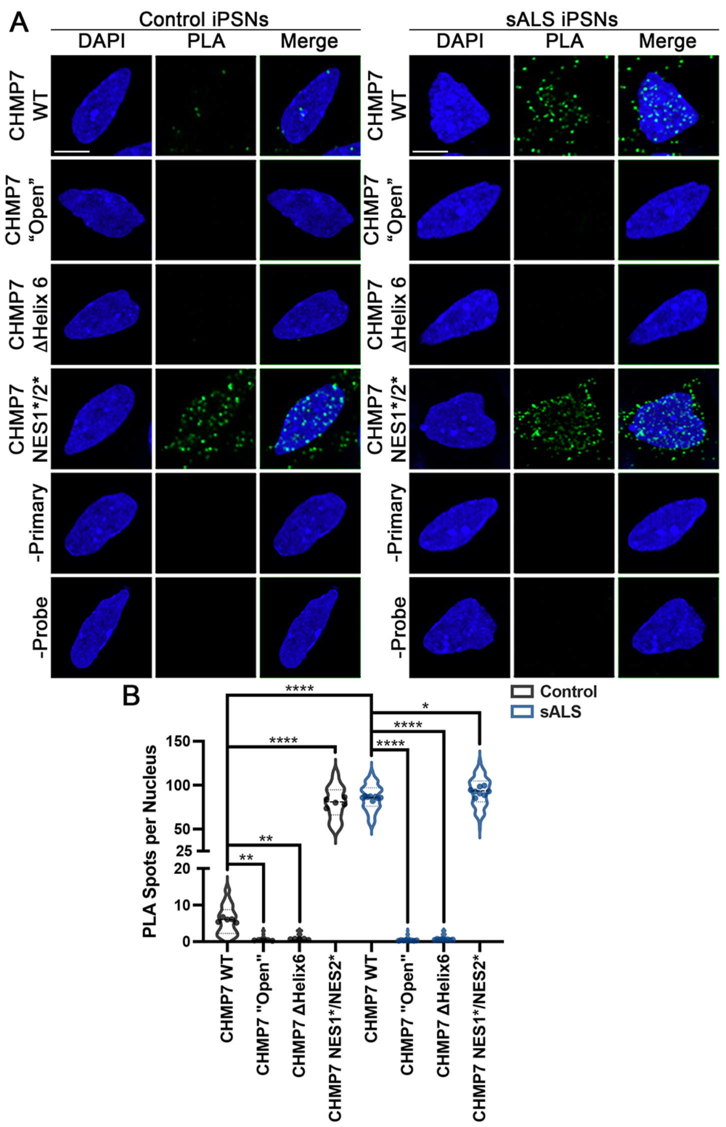

Supplemental Figure 15: CHMP7 "Open" and CHMP7 ΔHelix 6 mutants do not associate with CHMP2B in iPSNs. (**A**) Maximum intensity projections from confocal imaging of Flag – CHMP2B PLA signals in control and sALS iPSNs. CHMP7 plasmid as indicated on left, genotype as indicated on top. Note: Anti-Flag antibodies were used to detect Flag tagged CHMP7 proteins. (**B**) Quantification of number of PLA signals per nucleus. n = 5 control and 8 sALS iPSC lines, at least 100 nuclei per line/plasmid. Two-way ANOVA with Tukey's multiple comparison test was used to calculate statistical significance. * $p < 0.05$, ** $p < 0.01$, **** $p < 0.0001$. Scale bar = 5 μm.
